# *Oryza sativa* Cytochrome P450 Family Member OsCYP96B4 Reduces Plant Height in a Transcript Dosage Dependent Manner

**DOI:** 10.1371/journal.pone.0028069

**Published:** 2011-11-28

**Authors:** Rengasamy Ramamoorthy, Shu-Ye Jiang, Srinivasan Ramachandran

**Affiliations:** Rice Functional Genomics Group, Temasek Life Sciences Laboratory, National University of Singapore, Singapore, Singapore; French National Centre for Scientific Research, Université Paris-Sud, France

## Abstract

**Background:**

Plant cytochromes P450 are involved in a wide range of biosynthetic reactions and play various roles in plant development. However, little is known about the biological functions of the subfamily CYP96 in plants.

**Methodology/Principal Findings:**

Here, we report a novel semi-dwarf rice mutant, in which a single copy of transposon *dissociator* (*Ds*) was inserted into the gene *OsCYP96B4* (*Oryza sativa Cytochrome P450 96B4*). The mutant exhibits the defects in cell elongation and pollen germination, which can be complemented by the wild type *OsCYP96B4* and be rescued by remobilization of the *Ds* element with the presence of the transposase *Activator* (*Ac*). Transgenic plants harboring *OsCYP96B4* double-stranded RNA interference construct mimicked the mutant phenotype. The *oscyp96b4* mutant phenotype could not be rescued by all the tested phytohormones and it was found that *OsCYP96B4* reduced plant height in a transcript dosage dependent manner. Heterologous expression of *OsCYP96B4* in *Schizosaccharomyces pombe* resulted in missegregation and wider cells. Further investigation showed that the mutant exhibited the defects in the metabolism of some lipid molecular species when compared with the wild type.

**Conclusions/Significance:**

The *oscyp96b4* mutant is a novel rice semi-dwarf mutant. Our data suggest that *OsCYP96B4* might be involved in lipid metabolism and regulate cell elongation.

## Introduction

Dwarfism and semi-dwarfism are valuable agronomic traits in crop breeding. The semi-dwarf rice variety Dee-geo-woo-gen from China is well known as the parent in developing the semi-dwarf high yielding variety IR8, a leader of the green revolution, hence named green revolution rice [1]. The mutation in *SD1* gene, which encodes gibberellin 20-Oxidase, causes the semi-dwarf phenotype due to alteration in gibberellin level [1].

Various factors may contribute to dwarfism in plants. Over-expression, knockout or knockdown of different genes may result in reduced plant height. These genes are known to encode various proteins including tyrosine decarboxylase [2], cellulose synthase-like D (CSLD) [3], [4], a GDSL lipase superfamily member [5], a kinesin-4 protein [6], or a germin-like protein1 [7]. Molecular genetic and biochemical studies have revealed that gibberellins (GA) and brassinosteroid (BR) are the most important factors in determining plant height [8]–[13]. Many BR and GA-deficient or insensitive mutants show the dwarf phenotype [14]–[22]. Some of the affected genes were involved in plant growth hormone biosynthesis. Among them are genes encoding cytochrome P450 monooxygenases (CYPs). For example, the dwarf-related genes *D2*, *D35* and CYP90C1/D1 encode a brassinosteriod C-3 oxidase [16], an *ent*-kaurene oxidase [23] and BR C-23 hydroxylases [24], respectively. CYPs are a family of membrane-bound heme-containing proteins, found in both eukaryotic and prokaryotic organisms. These proteins mediate a wide range of redox reactions involved in the biosynthesis of plant hormones and secondary metabolites including synthesis of gibberellins (GA), brassinosteroid (BR), lignins, UV protectants, pigments, defense compounds, fatty acids, and signaling molecules.

CYPs constitute one of the largest super gene families in rice, consisting of 455 members and the functions of which are still unknown for most of them [25]. These genes were classified into 10 clans, four of which, have multiple gene families namely CYP71, CYP 72, CYP85 and CYP86 and the remaining six clans consist of single family including CYP51, CYP74, CYP97, CYP710, CYP711 and CYP727 [25]. Among the 10 clans of P450s, we were interested in the clan CYP86, which consists of seven families namely 86, 94, 96, 704, 730, 731 and 732. Functions of the clan CYP86 families are being investigated especially for the CYP86A sub-family. Such studies revealed that this sub-family functions as fatty acid ω-hydroxylases [26]–[30] but functions of other sub-families have yet to be determined. The CYP96 family in the clan CYP86 has eight sub-families namely CYP96A, CYP96B, CYP96C, CYP96D, CYP96E, CYP96F, CYP96G and CYP96H. The CYP96A sub-family comprises of fifteen genes in *Arabidopsis thaliana* and sub-family CYP96B has twelve genes and are found in four different plant species including oats (*Avena sativa*), rice (*Oryza sativa*), *Catharanthus roseus*, and black cottonwood (*Populus trichocarpa*) [25]. The functions of this sub-family are still unknown. In periwinkle (*Catharanthus roseus*), *CYP96C1* transcripts were accumulated only in alkaloid-producing cells and were induced for terpenoid indole alkaloid production [31]. In Arabidopsis, one of the CYP96A subfamily members, CYP96A15, has been reported to function as a mid-chain alkane hydroxylase in fatty acid hydroxylation [32]. No other data is available for this sub-family.

In this present study, we report the identification and characterization of a semi-dwarf mutant with dark green leaves and reduced grain yield from a visible *Ds* insertion line screen. In this mutant, *Ds* element was inserted into a *CYP* gene, designated as *OsCYP96B4* according to the P450 nomenclature. Our data show that the semi-dwarf phenotype is due to retardation in cell extension and the gene may reduce plant height by a transcript dosage dependent manner.

## Results

### The *oscyp96b4 Ds* insertion line is a novel rice semi-dwarf mutant

**Figure 1:**
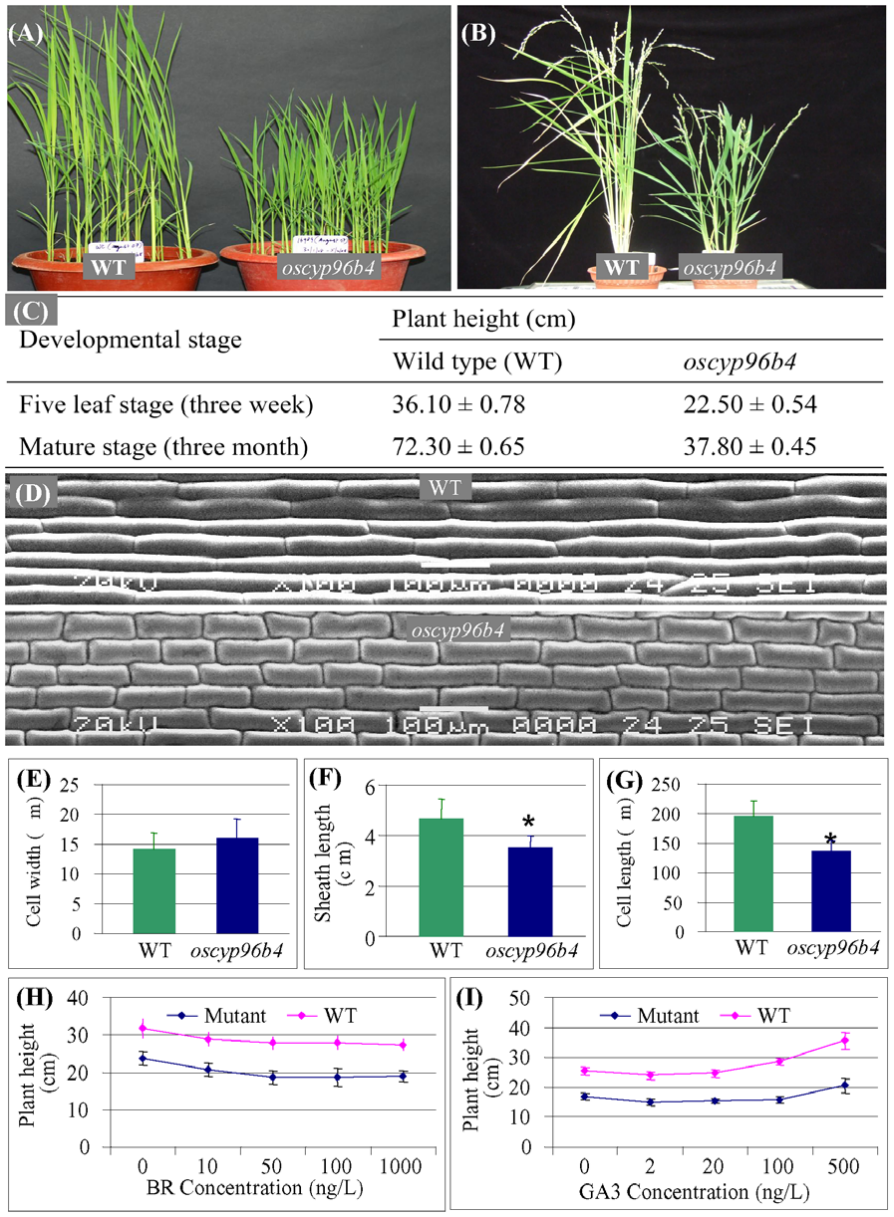
Phenotypic characterization of *oscyp96B4* as a dwarf mutant. (A) and (B) Three-week old seedlings and mature plants of WT (left) and *oscyp96b4* (right), respectively. (C) Detailed measurement of plant heights at both stages in WT (left) and *oscyp96b4* (right). These stages include five-leaf stage (three week old) and mature stage (three month old). (D) Cryo-SEM images of 2^nd^ leaf sheath surface of WT (top) and *oscyp96b4* (bottom). Bars = 100 μm. (E) to (G) show the results from the investigation on cell width (E), sheath length (F) and cell length (G). (H) and (I) show the effect of BR (H) and GA3 (I) on the plant height in WT (blue) and *oscyp96b4* (pink). Asterisks in (F) and (G) indicate significant differences between WT and the mutant plants at P<0.05 by *t*-test.

We have generated a large collection of around 20,000 *Ds* insertion lines [33], [34]. Of these, one mutant whose height was around two thirds of the wild type (WT) was selected for further characterization (Figure 1A). The difference in plant height was consistent throughout the vegetative growth and was also observed at the mature stage (Figure 1B). At the five-leaf stage (one month old), this semi-dwarf mutant was around 22.5 cm with about 38% reduction in plant height when compared with WT plants and at the mature stage, the mutant was around half the height of WT plants (Figure 1C). To analyze whether the observed semi-dwarf phenotype was due to a cell division or elongation defect, leaf sheath cells were analyzed by cryo-scanning electron microscopy. The mutant cells were shorter in length when compared with WT (Figure 1D). The images also showed that the cell numbers of the mutant leaf sheath were slightly more but not statistically different when compared with the WT. The width of the mutant cell was also slightly more than WT, with no significant difference as found out by statistical analysis (*t*-test, p>0.05, Figure 1E). However, the leaf sheath length showed significant difference between WT and the mutant (*t*-test, p<0.05, Figure 1F). The average cell length of the mutant was around 30% less (*t*-test, P<0.05) when compared with the WT (Figure 1G). These data suggest that the prolonged reduction of cell length throughout plant growth could have resulted in the decrease in plant height of the mutant.

Since dwarf phenotype is usually due to the changes in GA or BR metabolisms, we further investigated whether the semi-dwarf phenotype is due to a defect in endogenous hormone levels. Overall, with the increasing concentration of exogenous brassinolide (a synthetic form of BR), both WT and mutant showed inhibition in plant growth (Figure 1H) and increasing concentration of GA_3_ resulted in increase of plant height in both WT and mutant seedlings, respectively (Figure 1I). Thus, o*scyp96b4* did not show any pronounced effect or sensitivity to the hormone treatments when compared with WT grown under similar conditions. Furthermore, when WT and mutant seedlings were treated with other hormones including abscisic acid (ABA), indole-3-acetic acid (IAA), kinetin (KT), methyl-jasmonate (MeJA), and salicylic acid (SA), there were no significant difference between WT and the mutant (*t*-test, p>0.05, Figure S1). However, we still cannot exclude the possibility that OsCYP96B4 may be involved in phytohormone metabolism without testing the endogenous hormone levels.

### The *oscyp96b4* mutant shows the defect in pollen germination

**Figure 2:**
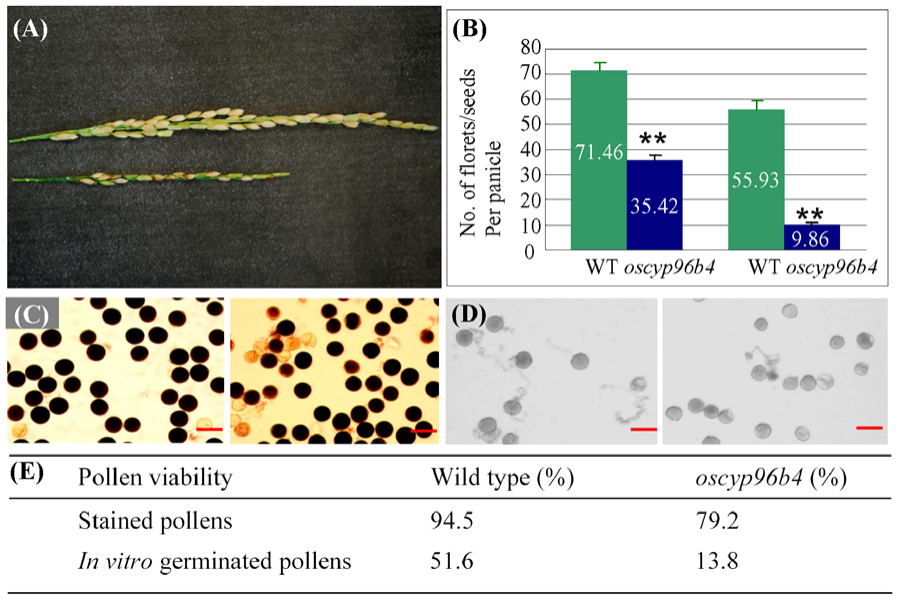
Defects of *oscyp96B4* at the reproductive stage. (A) Panicles in WT (top) and mutant (bottom). (B) Florets and seeds in WT (green) and mutant (blue). The symbols “**” indicate significant differences between WT and the mutant plants at P<0.01 by *t*-test. (C) Pollens stained with I_2_/KI in WT (left) and mutant (right). Starch can be stained by the I_2_/KI solution and the starch content in pollen grains can serve as an indicator of viability. (D) Germinated pollens in WT (left) and mutant (right). Germination rate of pollens in WT is higher than that in the mutant. (E) Pollen viability in WT and mutant. Bars in (C) and (D) = 50 μm.

The mutant not only showed reduced plant height but also exhibited defects in panicle development with significantly shorter panicle length when compared with WT (Figure 2A). The mutant also developed around 50% lesser number of florets and significantly lower viable seeds per panicle compared to the WT plants (*t*-test, p<0.01; Figure 2B). The lower seeding rate was due to the defect in male but not female reproductive organs as found out by the reciprocal sexual hybridization. We further examined pollen viability and tube growth by iodine/potassium iodide (I_2_/KI) staining and *in vitro* germination, respectively. Upon I_2_/KI staining for starch content, most of the pollen was stained in both the mutant and WT, respectively (Figure 2C). The WT and mutant pollen grains were then germinated *in vitro*; it was found that the rate of germination of the mutant was lower than the WT (Figure 2D). In the WT, 94.5% of pollens could be stained and 51.6% of them could germinate *in vitro* (Figure 2E). In the mutant, 79.2% of pollens could be stained; however, only 13.8% of them could germinate (Figure 2E). The data suggest that the reduced seeding rate in the mutant could be due to the defect in pollen germination.

### *Ds* was inserted into *OsCYP96B4* encoding a member of the cytochrome P450 multi-gene family in rice

To further investigate the molecular nature of the defect, we obtained the *Ds* Flanking Sequence tagged (FST) by TAIL-PCR and sequenced the amplified product. The sequence obtained was subjected to BLAST search using rice genome database which revealed that the *Ds* element had transposed into the coding region of a gene encoding cytochrome P450, designated as OsCYP96B4 according to the proposed nomenclature for P450 as it belongs to the CYP96 subfamily.

**Figure 3:**
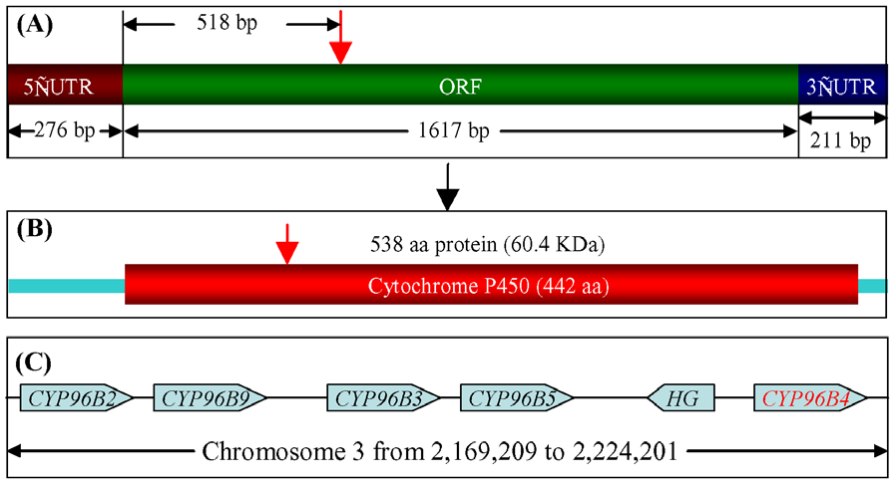
The genomic organization, structure and sequence analysis of *OsCYP96B4* and its protein. (A) Full-length cDNA of the *OsCYP96B4* gene showing various regions and the *Ds* insertion position as indicated by the red arrow. (B) The OsCYP96B4 protein showing the P450 domain (from Pfam database search; http://www.sanger.ac.uk/Software/Pfam/search.shtml) and the corresponding position of *Ds* insertion as indicated by the red arrow. (C) The tandemly duplicated 5 genes encoding the same class of P450 and their genome organization. The chromosome position of these genes were based on the release 6.1 of the Rice Pseudomolecules published by the Rice Genome Annotation Project (http://rice.plantbiology.msu.edu/index.shtml). HG, hypothetical protein.

*OsCYP96B4* contains a 1617 bp open reading frame flanking with 276 bp 5′-UTR and 211 bp 3′-UTR (Figure 3A). The gene is devoid of intron sequences and the encoded protein is 538 amino acids long, with an estimated molecular mass of 60.4 kDa. The *Ds* element was found to be inserted into the gene at 517^th^ base pair corresponding to the position between 172^nd^ and 173^rd^ amino acids of the protein sequence in 3′ to 5′ *Ds* orientation (Figure 3B). Thus, the *OsCYP96B4* gene should not be functional after the *Ds* insertion due to the breakdown of the functional domain. The subfamily has 9 members in rice (i.e. OsCYP96B2-OsCYPB10 [25]. Five of them (B2, 3, 4, 5 and 9) are tandemly localized on chromosome 3 (Figure 3C), suggesting tandem duplication as the major mechanism for the subfamily expansion.

### The semi-dwarf phenotype co-segregated with a single *Ds* insertion

The genomic DNA of WT and *oscyp96b4* were digested with five different restriction enzymes (*Cla*I, *Eco*RI, *Hin*dIII, *Kpn*I and *Pst*I), blotted on to a nylon membrane and hybridized with the β-Glucuronidase (GUS) gene probe. The result revealed that *oscyp96b4* had single copy *Ds* insertion (Figure S2A). Disruption of *OsCYP96B4* by *Ds* element was checked by hybridizing similar nylon membrane with *OsCYP96B4* 3′UTR region probe. The results showed a band shift between the WT and the mutant which confirmed the insertion of *Ds* element in the *OsCYP96B4* gene in the mutant genome (Figure S2B). To check if the mutant phenotype was linked to the *Ds* insertion, segregation analysis was performed. Homozygous mutants were crossed with WT plants and 15 F1 seeds were obtained. These seeds were germinated and F1 generation was obtained which were allowed to self-pollinate to obtain F2 seeds. A total of 188 F2 plants were analyzed for the segregation of both phenotypes and their genotypes. Of these plants, approximately, a 3:1 ratio of phenotype segregation was observed, i.e., 54 plants showed the dwarf phenotype and the rest were like WT, suggesting that the mutant was recessive (Figure S2C).

To determine the genotypes of these plants, PCR screening was done using three different primer sets specific to *BAR*, *GUS* and sequences flanking the *Ds* insertion site [33]. When the genomic DNAs from 54 plants which showed mutant phenotype were used as templates for PCR analysis, corresponding bands of right size were amplified using *BAR* and *GUS* specific primer sets, but no product was amplified from the flanking primer sets. The result indicates that all semi-dwarf plants are homozygous for the *Ds* insertion. Of the remaining 134 F2 plants, 46 plants were *BAR* and *GUS* PCR negative and showed positive bands for flanking primer-specific PCR, confirming that these plants were segregated nulls (WT). The remaining 88 plants were *GUS*, *BAR* and flanking primer-specific PCR positives indicating a heterozygous genotype. Thus, genetic analysis of *oscyp96b4* indicated that the mutation is recessive and the phenotype co-segregated with the *Ds* insertion.

### Revertants of the mutant showed the normal plant height and fertility

To confirm that the disruption of *OsCYP96B4* by *Ds* is indeed the cause for the mutant phenotype, reversion experiments were carried out by crossing homozygous mutant plants as female parent with the *Ac* transposase containing transgenic plants as the male donor. A total of 60 R1 seeds were obtained. From these seeds, R1 plants were generated and self-crossed to produce R2 seeds. Subsequently, the imbibed R2 seeds were screened for Green Fluorescent Protein (GFP) fluorescence and the seedlings devoid of fluorescence (negative selection for *Ac* transposase) were selected to check the reversion of the dwarf phenotype. A total of 735 such lines were selected to molecularly characterize the excision of *Ds* element. DNA fragments were amplified by PCR from genomic DNA samples isolated from these plants as templates using specific primers flanking the *Ds* insertion position. These PCR products were sequenced and upon analysis 39 plants showed footprints of varying sizes indicating the excision of *Ds* element from *OsCYP96B4*. We have observed three different footprints as shown in Figure S2D. The first two revertants showed one or eight base pairs of footprints. Thus, these revertant plants still produced non-functional or abnormal proteins due to the reading frame shift mutation by the footprints. The third revertant showed nine base pairs of footprints which add three amino acids in frame to the WT *OsCYP96B4* coding sequence. Thus, this revertant might produce normal and functional protein. Among three revertants, base substitutions were observed in all three footprints during *Ds* remobilization under the presence of *Ac* transposase (blue fonts in Figure S2D), which was one of the footprint forming mechanisms [35], [36].

We then investigated the phenotypes of these revertant plants. We observed that only one of the three revertant plants exhibited normal plant height and fertility like WT (Figure S2 E and F). This is the revertant which showed nine base pairs of footprints which added three amino acids in frame to the WT *OsCYP96B4* coding sequence. The remaining two revertants still showed the semi-dwarf phenotype and abnormal pollen development due to the reading frame shift mutation (Figure S2E). This analysis confirmed that the observed mutant phenotype was due to the *Ds* insertion into the coding region of the *OsCYP96B4* gene.

### *OsCYP96B4* genetically complements the mutant phenotype

As an additional confirmation to the revertant analysis, genetic complementation of *oscyp96b4* was carried out. The genomic region of *OsCYP96B4* driven by its own promoter fragment (a 2 kb fragment upstream of the start codon) was cloned and transformed into the homozygous mutant plant. In this construct, around 1 kb of 3′ end of genomic sequences including the translation termination codon of *OsCYP96B4* was also included. A total of 5 independent transgenic plants harboring this construct in the mutant background were selected with single copy of T-DNA insertion according to Southern blotting analysis (Figure S2G). These transgenic plants showed normal plant heights in T0 and T1 generations when compared with the WT plant (Figure S2H). The expression level of *OsCYP96B4* in these complemented plants is similar to that in WT as found out by qRT-PCR analyses (Figure S2I). These plants also exhibited normal fertility. The result further confirmed that the mutant phenotypes of *oscyp96b4* were caused by *Ds* element insertion into the *OsCYP96B4* gene.

### *OsCYP96B4* was expressed in all tested tissues with differential abundance

**Figure 4:**
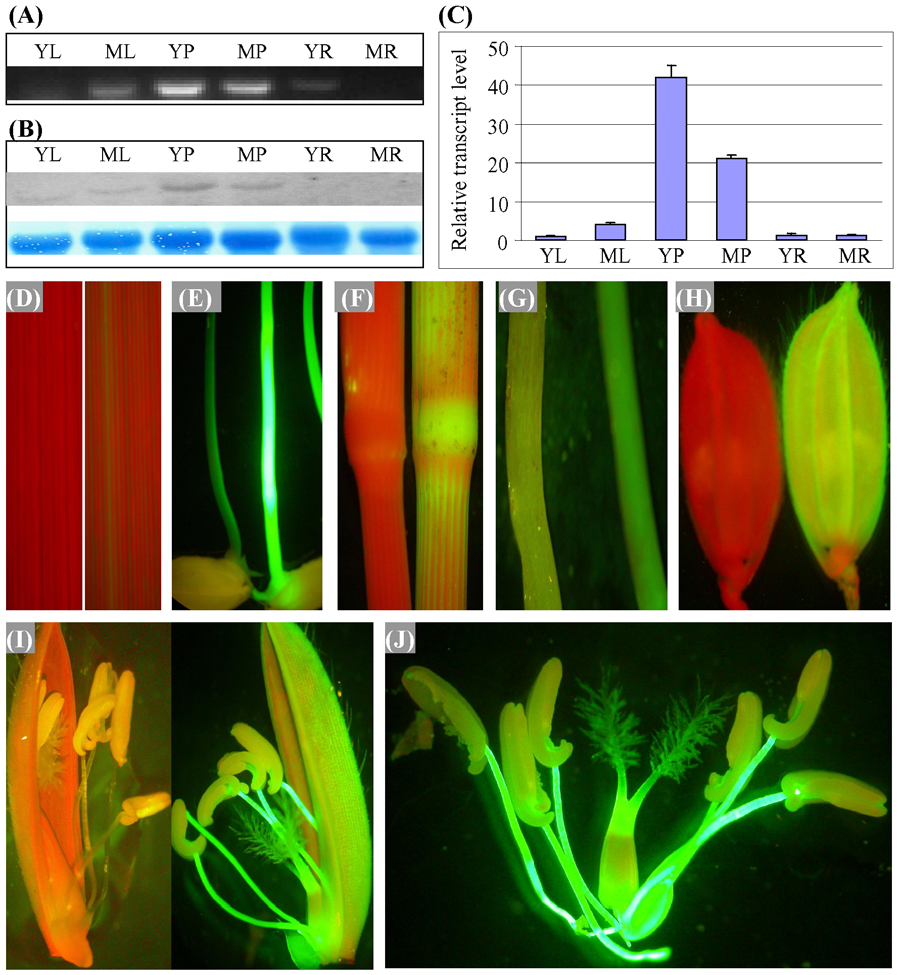
Expression profiles of *OsCYP96B4* in rice. The expression analysis was carried out by RT-PCR (A) and was verified by Northern bloting (B) and qRT-PCR (C). In (A) to (C), YL, ML, YP, MP, YR and MR indicate young leaf, mature leaf, young panicle, mature panicle, young root and mature root, respective. (D) to (J) show the expression patterns based on the fluorescent analysis of the *OsCYP96B4* promoter-GFP transgenic plants. In (D) to (I), left and right images were taken from WT and the *OsCYP96B4* promoter-GFP transgenic plants, respectively. (D) Two-week old leaves; (E) Geminated seeds; (F) Stem nodes; (G) Roots from two-week old seedlings; (H) Florets from nonflowering panicles; (I) and (J) Opened florets.

Despite the fact that the gene trapped *GUS* gene within the *Ds* element was in the right orientation, the expression of glucuronidase could not be detected in the mutant plants due to the reading frame shift in the fusion region. To study the expression pattern of this gene, RT-PCR analysis were carried out and the data revealed that *OsCYP96B4* was ubiquitously expressed in all tissues tested (Figure 4A). However, the gene exhibited differential transcript abundance at different tissues with the highest expression in the reproductive tissues i.e., in young and mature panicles and the lowest level in young and mature roots (Figure 4A). Such expression patterns were also confirmed by Northern blotting and qRT-PCR analyses (Figure 4 B and C).

To check the expression of this gene at the cellular level, a synthetic *Green Fluorescent Protein* (*sGFP*) was cloned and was driven by the endogenous *OsCYP96B4* promoter. The construct was then introduced into the WT genome. The transgenic *OsCYP96B4*-promoter-*GFP* plants harboring this construct were subjected to fluorescence microscopy analysis. The result showed that the promoter activity was weak in leaves (Figure 4D). Higher GFP activities could be detected in germinated shoots (Figure 4E), stem nodes (Figure 4F) and roots (Figure 4G). Interestingly, the promoter showed the expression in the whole florets (Figure 4H) with the highest activity in the anther filaments (Figure 4 I and J).

### Transgenic plants harboring *OsCYP96B4* double-stranded RNA interference construct mimick the mutant phenotype

**Figure 5:**
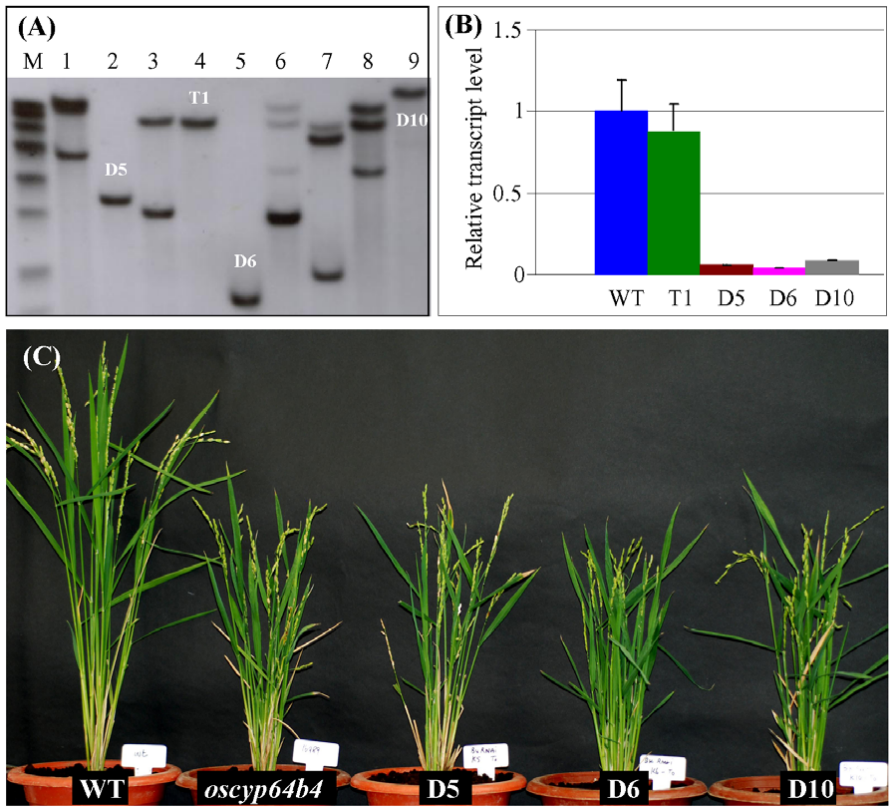
Double strand RNAi-mediated specific silencing of *OsCYP96B4* in rice. (A) Determination of T-DNA copy numbers by Southern blot hybridization. (B) Expression analysis by qRT-PCR in various transgenic plants. In (A) and (B), T1 indicates the transgenic plant with the empty T-DNA insertion as a control; D5, D6 and D10 indicate the double strand RNAi transgenic plants with single copy of T-DNA insertion. (C) Phenotypic characterization of three independent dsRNAi transgenic plants by compared with WT and mutant.

After *Ds* insertion into *OsCYP96B4*, the gene still encodes a fusion protein containing the first 172 aa of the protein OsCYP96B4 (one third of the protein). To investigate whether this truncated protein is functional or not, we made a double-stranded RNA interference (dsRNAi) construct. A unique region of 525 bp from 5′untranslated leader sequence plus coding region of *OsCYP96B4* were PCR amplified and cloned into a dsRNAi vector, pTCK303. The dsRNAi construct was then introduced into WT by *Agrobacterium*-mediated transformation. Four independent lines with single copy of T-DNA insertion were selected by Southern blot hybridization for further analysis (Figure 5A). These lines were designated as D5, T1, D6 and D10. Endogenous *OsCYP96B4* expression analysis showed that the *OsCYP96B4* mRNA level was similar to WT in the T1 line whereas the *OsCYP96B4* transcript abundance was dramatically decreased (at least fourfold) in the remaining 3 lines including D5, D6 and D10 (Figure 5B). The data suggest that the dsRNAi functions in the D5, D6 and D10 lines but not in the T1 line. As a result, no significant difference in its phenotype was observed in the T1 transgenic line (*t*-test, p>0.05). On the contrary, the D5, D6 and D10 lines mimicked the *oscyp96b4* mutant phenotype due to the expression suppression by dsRNAi (Figure 5C). It is suggested that the truncated protein formed due to *Ds* insertion might not be functional and confirmed that *OsCYP96B4* played a role in controlling plant height.

### *OsCYP96B4* controls plant height in a transcript dosage dependent manner

**Figure 6:**
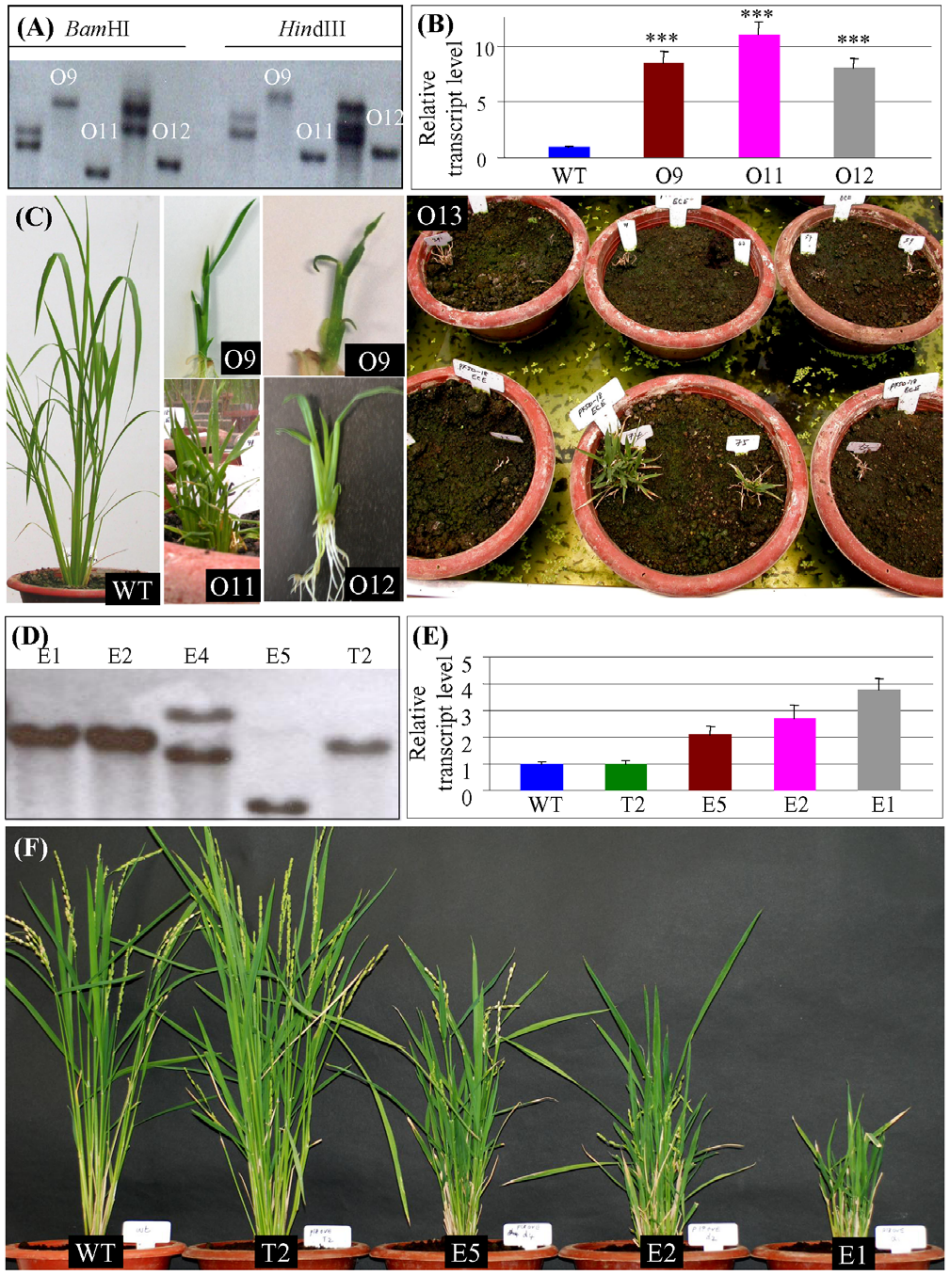
Phenotypic analysis of transgenic plants by over- and ectopic expressing *OsCYP96B4*. (A) Different patterns of T-DNA integration in transgenic plants harboring *OsCYP96B4* driven by the maize ubiquitin promoter in WT (over-expression). (B) Bar diagrams showing the expression level of *OsCYP96B4* in WT and three independent over-expression dwarf plants with single copy of T-DNA integration. The symbols “***” indicate significant differences in their expression level between WT and the transgenic plants at P<0.001 by *t*-test. (C) Phenotypic characterization of over-expression transgenic plants. O9, O11 and O12 showed severe dwarf phenotype in different transgenic plants. O13 shows that most of transgenic plants died after several months of growth. (D) Different patterns of T-DNA integration in transgenic plants harboring *OsCYP96B4* driven by its own promoter in WT. (E) Bar diagrams showing the expression level of *OsCYP96B4* in WT and three independent ectopic expression plants (E1, E2 and E4) as well as transgenic plants with the empty T-DNA integration (T2). The symbols “*”, “**” and “***” indicate significant differences in their expression level between WT and the transgenic plants at P<0.05, P<0.01 and P<0.001, respectively, by *t*-test. (F) Phenotypic characterization of ectopic expression transgenic plants by comparing with WT and T2 plants. In (A) and (D), the results are based on Southern blot hybridization.

To further investigate and understand the effect of *OsCYP96B4* on plant height, transgenic plants over-expressing the *OsCYP96B4* gene under the control of strong constitutive maize ubiquitin promoter were generated. DNA samples from total of five T0 transgenic plants were analyzed by Southern blotting to determine the copy number of T-DNA insertion (Figure 6A). Among them, three independent transgenic lines with the single T-DNA insertion were selected for further analysis and they were named as O9, O11 and O12. The qRT-PCR analysis showed that the expression level of *OsCYP96B4* was significantly increased in all the three tested independent lines when compared with WT (*t*-test, p<0.001; Figure 6B). Phenotype investigation showed that all of three lines exhibited severely stunted growth (Figure 6C, O9, O11 and O12). Most of them grew at a slow rate and did not flower; as a result, most of these transgenic plants died after several months of vegetative growth (Figure 6C, O13). The expression abundance and phenotype analysis showed that the expression dosage of *OsCYP96B4* has an effect on plant height.

Since the higher expression level of *OsCYP96B4* by over-expression resulted in very short and clustered plants with severely stunted growth, transgenic plants ectopically expressing *OsCYP96B4* were generated by introducing the *OsCYP96B4* gene under the control of its own promoter into WT to investigate the effect of expression dosage on plant height. Three independent transgenic lines with single copy of *Ds* insertion were selected for further analysis and they were named as E1, E2 and E5 (Figure 6D). RNA samples were isolated from these transgenic plants and were then subjected to qRT-PCR to examine the transcript level of *OsCYP96B4*. The WT and T2 plants with the empty T-DNA insertion were used as controls. The expression analysis showed that three lines E1, E2 and E5 exhibited higher *OsCYP96B4* transcript abundance than both WT and T2 transgenic plants (Figure 6E). Among these three lines, E5 showed the lowest expression level and E1 showed the highest transcript abundance whereas expression of E2 was intermediate to that of E5 and E1 (Figure 6E). Their phenotypes were then characterized in T0 and T1 generations. Transgenic plants expressing the *OsCYP96B4* transcript showed different phenotypes with varying plant heights including severe dwarf, semi-dwarf (which were similar to the *oscyp96b4* mutant) and normal WT height (Figure 6F). The *OsCYP96B4* transcript and phenotypic characterization further confirmed the transcript level-dependent effect of this gene on plant height i.e., the mRNA levels of *OsCYP96B4* was higher in those lines which mimicked the mutant phenotype, whereas the levels were lower in the plants which resembled the WT plants. In contrast, the lines with severe dwarf phenotype had the highest amounts of *OsCYP96B4* transcript levels (Figure 6 E and F). These results revealed that *OsCYP96B4* expression is strictly regulated and plant height varies in a dosage dependent manner.

### Over-expression of *OsCYP96B4* in Arabidopsis reduced leaf size and the length of anther filaments

To better understand the role of *OsCYP96B4*, transgenic Arabidopsis plants were generated by over-expressing this gene and their phenotypes were investigated. Our data showed that the transgenic Arabidopsis leaves exhibited reduced size (Figure S3A). On the other hand, over-expression of *OsCYP96B4* in Arabidopsis also significantly reduced the length of anther filaments (Figure S3B), suggesting the role of this gene in cell elongation. The most closely related members of *OsCYP96B4* in Arabidopsis are *AtCYP96A1* and *AtCYP96A10* as found out by BLAST searches and phylogenetic analysis. T-DNA insertion mutants of these two genes in Arabidopsis were obtained from the Arabidopsis Biological Resource Center (http://abrc.osu.edu/). However, T-DNA insertion mutants of these two genes in Arabidopsis did not show a similar phenotype, i.e. reduced plant height or size. These T-DNA insertion lines showed similar phenotypes to WT plants and they exhibited similar plant heights and silique development (Figure S3 C-D). These data suggest that homologous genes of *OsCYP96B4* in Arabidopsis might have evolved into different biological functions.

### Heterologous expression of *OsCYP96B4* in *Schizosaccharomyces pombe* resulted in mis-segregating and wider cells

**Figure 7:**
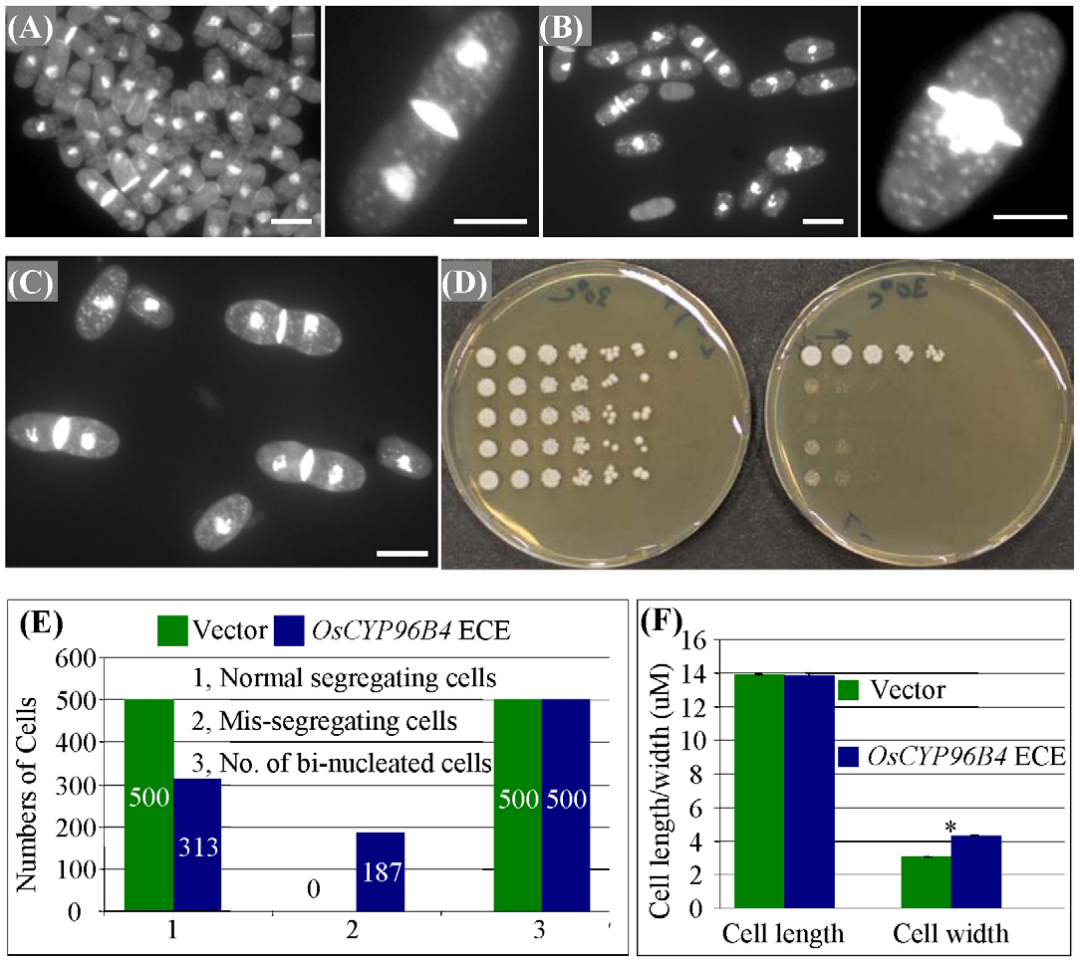
Heterologous ectopic-expression of *OsCYP96B4* in *S. pombe*. (A) A group of *S. pombe* cells expressing the vector control (left) and a magnified view of single vector control cell (right). (B) A group of cells showing ectopic expression of *OsCYP96B4* in the yeast cells with chromosome segregation defect (left) and a magnified view of ‘cut’ phenotype. (C) The yeast cells ectopically expressing *OsCYP96B4* show dumbbell shape. (D) Over-expression of *OsCYP96B4* in the yeast is toxic to its cells. The left shows the control and the right is the overexpressor. (E) The effects of ectopic expression of *OsCYP96B4* in the yeast on cell segregation. (F) An investigation of cell length and width of the *S. pombe* cells upon ectopic-expression of *OsCYP96B4*. Bars in (A) to (C) = 5 μm. The asterisk in (F) indicates the significant difference between the control and ectopic expression of *OsCYP96B4* in yeast at P<0.05 by *t*-test.

To check the effect of OsCYP96B4 protein on cell morphology, we ectopically expressed *OsCYP96B4* gene in another model organism, fission yeast (*S. pombe*), where its polarity is well defined. The fission yeast cells with the control vector are cylindrical in shape, grow by tip extension and divide by medial fission (Figure 7A). However, over-expression of *OsCYP96B4* in yeast led to defects in chromosome segregation (Figure 7B) and to the formation of dumbbell shaped cells (Figure 7C). Over-expression of *OsCYP96B4* was also toxic to the yeast cells (Figure 7D).

In the yeast expressing control vector, all the observed 500 cells showed normal segregation, none of them showed missegregation and all of them were bi-nucleated cells (Figure 7E). However, in the yeast expressing *OsCYP96B4*, only 313 showed normal segregation and the remaining 187 exhibited missegregation cells (Figure 7E). On the other hand, although the cell length showed no difference between the control and *OsCYP96B4*-expressing yeast, the cell width was measured with higher value (*t*-test, p<0.05) due to the ectopic expression of *OsCYP96B4* in the yeast (Figure 7F).

### Differential lipid profiling between WT and the *oscyp96b4* mutant

**Figure 8:**
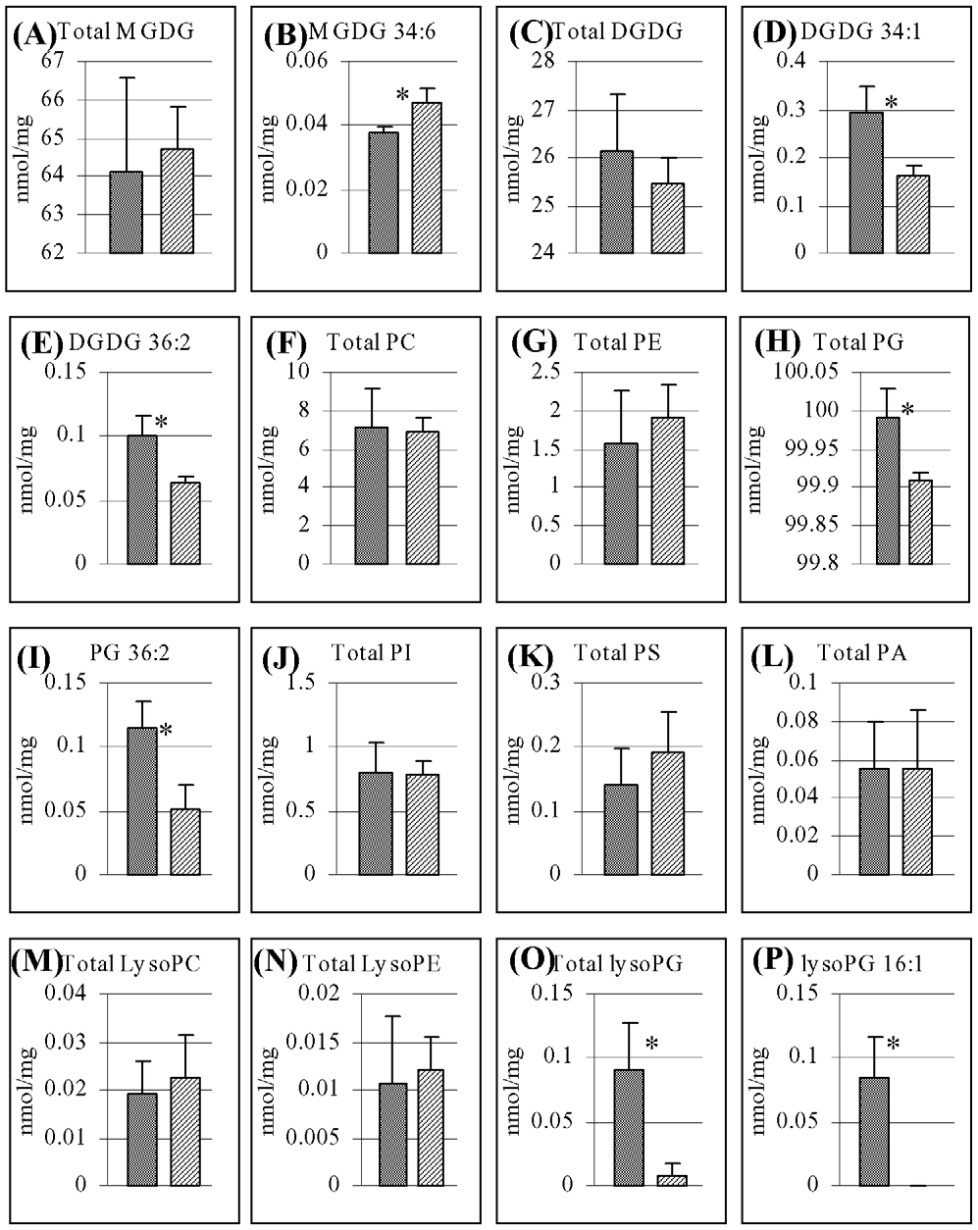
Differential lipid profiling between WT and *oscyp96b4*. (A) to (E) indicated the amount of total plant glycolipids MGDG and DGD and their specific molecular species. (F) to (K) showed the amount of total membrane phospholipids PC, PE, PG, PI and PS as well as their specific species. (L) to (P) showed the amount of total minor membrane lipid metabolites PA, lysoPC, lysoPE and lysoPG as well as their specific species. Only the molecular species with significant differences in their amount between WT and *oscyp96b4* are presented. The symbol “*” indicates the significant differences between WT and the *Ds* insertion mutant by the *t*-test statistic analysis at P<0.05. In (A) to (P), left and right columns indicate WT and *oscyp96b4*, respectively.

Total lipids extracted from *oscyp96b4* and WT were subjected to gas chromatography and mass spectroscopy analysis. We have analyzed the plant glycolipids namely monogalactosyldiacylglycerol (MGDG) and digalactosyldiacylglycerol (DGDG). No significant difference was detected between WT and the mutant in total either MGDG or DGDG (*t*-test, P>0.05); however, we detected significant differences in one or two molecular species (*t*-test, P<0.05; Figure 8 A–E). We then analyzed the common membrane phospholipids classes including phosphatidylcholine (PC), phosphatidylethanolamine (PE), phosphatidylglycerol (PG), phosphatidylinositol (PI) and phosphatidylserine (PS) (Figure 8 F-K). Our data showed that the mutant exhibited significant reduction in PG content either in total or in a specific species PG 36:2 (*t*-test, P<0.05; Figure 8 H and I We also analyzed minor membrane lipid metabolites, including phosphatidic acid (PA), lysophosphatidylcholine (lysoPC), lysophosphatidylethanolamine (lysoPE) and lysophosphatidyl-glycerol (lysoPG) (Figure 8 L-P). This analysis revealed reduced level of lysoPG in the mutant either in total amount or in a specific species 16:1 (*t*-test, P<0.05; Figure 8 O and P). All these data suggested that *OsCYP96B4* might be involved in plant lipid metabolism.

## Discussion

### Dwarf mutant phenotype and phytohormone metabolism

Till date, a great number of dwarf mutants have been identified in rice and some of these mutants have been further characterized with respect to the underlying genes. The analyses showed that various factors may lead to dwarfism. Many dwarf mutants were defective in the biosynthesis of phytohormone GA and BR as mentioned earlier. In contrast to these GA- and BR-related dwarf mutants, little is known about mutants that are defected in non-GA/BR-related pathways. In this study, we have identified a novel dwarf mutant. This mutant showed no difference in the response to both GA and BR when compared with WT plants. On the other hand, the expression of *OsCYP96B4* was also not regulated by GA or BR. In addition, the mutant phenotype cannot be rescued by spraying these two phytohormones. Thus, *oscyp96b4* seems to be a novel hormone independent mutant. Rice GA-related mutants show dwarf phenotype with dark green leaves but with no other abnormal morphology except for sterility in severely dwarf mutants [37]. In contrast, rice BR-related mutants show dwarf phenotype with other abnormal morphology, including malformed leaves [16]. The mutant *oscyp96b4* showed a similar phenotype to the typical GA-related mutant. However, our data showed that it is GA independent mutant. GA- or BR-related dwarf mutations usually have defects in hormone biosynthesis and metabolism, thereby affecting their cell elongation [38]. The dwarfism of the mutant *oscyp96b4* was also due to the reduced cell elongation (Figure 1), however, which was not resulted from defects in GA/BR biosynthesis. Currently, limited data is available for GA/BR independent mutants. The investigation of these mutants may provide novel insights into the mechanisms of dwarf phenotype. Recently, several investigations reported these GA/BR independent mutants and suggested new mechanisms of dwarfism [39], [40].

Since both GA and BR cannot rescue the dwarf phenotype, many other plant growth factors were also tested including auxins (IAA), cytokinins (KT), abscisic acid (ABA), methyl-jasmonate (MeJA), and salicylic acid (SA). However, none of them were able to rescue the phenotype. The data suggest the limited effect of *OsCYP96B4* on the metabolism of these tested hormones. However, further experiments should be carried out to examine if this gene may be involved in the hormone metabolisms by measuring endogenous hormone levels or testing other hormones.

### Both knockout/knockdown and over-expression of *OsCYP96B4* reduce plant height by decreasing cell elongation

Usually, knockout or knockdown of GA biosynthesis related genes may reduce plant height whereas over-expression of these genes may increase plant height. Similar results have been also reported on the roles of BR. In this study, manipulation of *OsCYP96B4* expression level could not make the plants taller than WT. When the *OsCYP96B4* gene was knocked out by *Ds* insertion, the mutant showed the semi-dwarf phenotype (Figure 1 A and B). Silencing of the gene by dsRNAi mimicked the mutant phenotype (Figure 5). The increased expression level by additional copy of the gene significantly reduced the plant height in a transcript dosage dependent manner (*t*-test, P<0.05; Figure 6 D-F). Since *OsCYP96B4* product is not a phytohormone, such as GA or BR biosynthesis related gene, no transgenic lines with increased plant height could be generated after increasing or decreasing the expression abundance of this gene. On the other hand, *OsCYP96B4* also functioned in Arabidopsis. Phenotypic investigation of transgenic Arabidopsis plants over-expressing this gene suggested a similar role in cell elongation (Figure S3). However, T-DNA insertion mutants of two homologous genes in Arabidopsis namely *AtCYP96A1* and *AtCYP96A10* showed no visible phenotypic variation when compared with the WT Arabidopsis plants (Figure S3). These data suggested that the homologous genes in Arabidopsis had limited effect on cell elongation and might have diverged in their biological functions during the long evolutionary period.

### *OsCYP96B4* in rice might play a role in lipid modification

CYP86A1, CYP86A2, CYP86A8, CYP86B1, CYP704B1 and CYP704B2 encode fatty acid ω-hydroxylases, and function in cutin and/ or suberin biosynthesis [27], [27,41]. The *OsCYP96B4* belongs to the clan CYP86 family of genes, from which a few genes have been reported to encode fatty acid hydoxylases [26], [27], [41] and one gene from a homologous gene family in *Arabidopsis CYP96A15* was recently reported to encode mid chain alkane hydroxylase [32]. Wellesen et al (2001) showed that the dwarfism observed in the *Arabidopsis lacerata* mutant defective in a cytochrome P450 monooxygenease (with a medium chain fatty acid hydroxylase function) could be due to the defect in the cutin biosynthesis leading to organ fusion [27]. Since our mutant shows semi-dwarf phenotype and *OsCYP96B4* belongs to the same clan (CYP86) similar to *LACERATA, OsCYP96B4* may be involved in modification of fatty acids. The heterologous expression of *OsCYP96B4* in *S. pombe* led to erroneous segregation of DNA (Figure 7). The phenotype is similar to the well characterized *cut6* mutant, where the mutated gene encodes acetyl CoA carboxylase, a key enzyme for fatty acid synthesis in *S. pombe* [42]. In addition, there was a formation of dumbbell shaped cells in the *OsCYP96B4* over-expressed cell populations that resembled a mutant called *lsd1* [43], and *LSD1* is known to encode a fatty acid synthetase. All these data suggested the involvement of *OsCYP96B4* in fatty acid modification. Interestingly, similar observations with respect to cell length and width were observed in the *oscyp96b4* mutant leaf sheath cells suggesting a possible role of this gene in fatty acid metabolism. Furthermore, the GC/MS analysis of total lipid revealed statistical differences between WT and the *oscyp96b4* mutant in several lipid molecular species (Figure 8), suggesting the involvement of this gene in lipid metabolism. However, it is still unknown how the changed lipid metabolism affects plant height and fertility.

### Sequence divergence of tandemly duplicated *CYP96* genes in plants

In rice, a total of 9 CYP96B sub-family members have been identified [25] and 5 of them are located on chromosome 3 with tandem array, suggesting the tandem duplication as the major mechanism for the expansion of this subfamily. In *Sorghum bicolor* and *Brachypodium distachyon*, tandemly duplicated CYP96B sub-family members were also found in related syntenic regions. They are Sb01g047600, Sb01g047610, Sb01g047630, Sb01g047640 in sorghum and Bradi1g75710, Bradi1g75730, Bradi1g75740 in *B. distachyon*. The most closely related member in the Arabidopsis genome is At4g39490, a CYP96A member. The CYP96A members on chromosome 4 were also tandemly duplicated. All this data suggested that tandem duplication of the CYP96 family occurred in the ancient era. However, phylogenetic analysis also showed that some of rice CYP96B members were tandemly duplicated after the divergence from sorghum (Figure S4A). Thus, we are wondering how these tandemly duplicated genes could survive during the relatively long evolutionary history. Amongst the 5 tandemly duplicated rice *CYP96B* members, amino acid sequence similarity ranges from 55–76% in their P450 domain regions (Figure S4B). These 5 members also showed obvious differences in their expression patterns (Figure 4 and Figure S4C). In fact, we have identified another mutant, where *Ds* was inserted into *OsCYP96B9*, with no significant difference in its plant height and other traits when compared with the WT plants. The data suggests that both sequence divergence and the difference in their expression patterns could be regarded as the possible driver for the retention of these duplicated genes.

## Materials and Methods

### Plant materials and growth conditions

The *Japonica* rice (*Oryza sativa* ssp japonica cv. Nipponbare) was used as the WT plants for all experiments. WT, *Ds* insertion lines and various transgenic plants were grown in greenhouse or in the field under natural light and temperature conditions.

### Visible phenotype screens

A large numbers of homozygous *Ds* transposants were planted in the green house and in the field for phenotype screens to identify mutant lines with altered morphology. These transposant lines were followed through from germination till mature stage. Various parameters such as seed germination, seedling growth, height, stature, panicle morphology, seed set etc., were scored during the plant development. This screen identified around 175 dwarf and semi-dwarf mutants of which an interesting semi-dwarf mutant was selected for further study.

### Genetics analysis of the *oscyp96b4* mutant

The *oscyp96b4* mutant was crossed with WT plants to obtain heterozygous seeds. The segregation population generated from heterozygous mutants was used for Basta resistance analysis and the phenotype investigation. The identification of genotypes at the *Ds* insertion locus was carried out by PCR analysis. A total of 188 individuals were used for segregation analysis.

### Rescue analysis of semi-dwarf mutant by phytohormones

Different concentrations (0, 0.01, 0.1, 0.5 and 1 µM) of GA_3_ and brassinolide (BL) were prepared with 100% ethanol and were added into MS media. MS media containing only the ethanol were used as a control. The homozygous mutant and WT seeds were germinated on these media and were grown at the 25–28°C with 16h light and 8h dark conditions for two weeks. Changes in the seedling morphology were monitored and height was measured. The measurements were subjected to statistical analysis. Rescue analysis with other phytohormones was also carried out according to the report [44]. The phytohormones used included auxin IAA, cytokinins KT, ABA, MeJA, and SA. All the hormones were added into MS media at different concentrations. ABA was prepared with Dimethyl Sulfoxide (DMSO) and MS media containing only DMSO were used as a control. The remaining hormones were prepared with 100% ethanol and ethanol containing media were used as controls. The concentrations used for ABA, IAA and KT were 0, 0.1, 1 and 10 µM. For MeJA, 0, 10, 50, 100 and 1000 nM were used; for SA, 0, 5, 10, 50 and 500 µM were used.

### Cryo-scanning electron microscopy (Cryo-SEM) and cell size determinations

The first and second leaf sheath of two-week-old WT and mutant seedlings were subjected to cryo-SEM to observe the differences in the cell file and size of epidermal cells. The leaf sheaths were frozen with liquid nitrogen. The samples were then mounted and the surface was scanned using Joel JSM-5310 LV electron microscope and the images were captured using JOEL SEMafore software (JOEL AB, Sundbyerg, Sweden). These images at 200X magnification were used to measure the cell length and width. Only the whole cells in the field of view were taken for such measurements. A total of 320 cells were measured and statistical analysis was performed to check the differences in the cell architecture.

### Reversion analysis

For reversion analysis, the homozygous mutant was crossed with transgenic sGFP-Ac plant carrying transposase [33]. GFP negative plants in F2 generation containing no Ac transposase were selected for further analysis. Genomic sequences flanking the *Ds* insertion site (footprint sequences) were obtained by PCR amplification followed by sequencing. The putative revertants were then planted and homozygous revertants were selected in the next generation for phenotype characterization.

### Genomic DNA and total RNA extractions, Southern and Northern blot analysis

Genomic DNA and total RNA preparation as well as Southern and Northern blot analyses were carried out according to the description by Jiang et al. (2005) [45]. Total RNA and mRNA samples were prepared by using Qiagen RNeasy Midi Kit (Cat.No. 75144) and QIAGEN Oligotex mRNA Midi Kit (Cat.No. 70042) according to the manufacturer's protocols. DIG-labeled *GUS* and *HPT* probes were synthesized using PCR DIG Probe Synthesis Kit (Roche Cat.No11636090910). The PCR products were purified by QIAquick gel extraction kit (QIAGEN Cat. No. 28706). Probe concentrations were determined using DIG labeled standard DNA (Roche) by serial dilution and probes were used as per manufacture recommendation. *GUS* and *HPT* probes were used to check the copy numbers of the *Ds* element and T-DNA insertions of transgenic plants, respectively.

For Southern blot hybridization, 5 µg of genomic DNA was digested with appropriate restriction enzymes, fractionated on 1.0% agarose gel and transferred onto a Hybond-N^+^ membrane (Amersham). DNA blots were hybridized with digoxigenin-labeled (DIG) probes in DIG Easy Hyb solution (Roche Cat.No. 11603558001) at 42°C. After hybridization, membranes were washed twice with 5× SSC and 0.1% SDS for 15 min, then twice with 0.5×SSC and 0.1% SDS for 15 min at 68°C and finally twice with 0.1×SSC and 0.1% SDS at 50°C. Detection was carried out according to the manufacturer's protocol using DIG Wash and Block Buffer Set and chemiluminescent substrate CDP-Star^TM^ (Roche Cat.No. 11685627001). Hybridization signals were visualized by autoradiography.

For Northern blot analysis, around 30 µg of total RNAs or in some cases 2 to 5 µg of mRNAs isolated from various tissues were fractionated on 1.2% agarose gel containing formaldehyde using BIORAD electrophoresis apparatus and were transferred onto a Hybond N^+^ membrane (Amersham). The blotted membrane was hybridized with DIG-labeled appropriate gene-specific probes in DIG Easy Hyb solution (Roche) at 50°C for overnight. Probes were synthesized by PCR with DIG Probe Synthesis Kit (Roche Cat.No. 11636090910). The post hybridization washes and signal visualizations were carried out as mentioned in Southern blot analysis.

### Molecular cloning of the *OsCYP96B4* gene and its promoter

The *OsCYP96B4* gene is devoid of intron and was amplified by PCR using genomic DNA as template. Gene or promoter-specific primers were selected for such amplification and the isolation of its promoter using Expand Long Template PCR system (Roche Cat.No. 11681842001) was carried out as per manufacturer's protocol. The primer sequences are listed in the Table S1. The PCR products were purified by QIAquick gel extraction kit (QIAGEN Cat. No. 28706) and were then cloned into pGEMT-Easy vector (Promega Cat. No. A1360). The cloned gene and its promoter were verified by restriction digestion and sequencing.

### Expression analysis by RT-PCR and qRT-PCR

All primers used for RT-PCR were selected using DNASTAR program and primers used for qRT-PCR were designed by Applied Biosystems Primer Express^®^ software. All primers used for both RT-PCR and qRT-PCR were submitted to NCBI database for BLAST searches to eliminate nonspecific primers. The primer sequences are listed in the Table S1. The first strand cDNA was synthesized from mRNA using Invitrogen SuperScript III First strand Synthesis System for RT-PCR (Cat.No. 18080-051). RT-PCR and qRT-PCR were carried our as described previously [34]. RT-PCR reactions were performed in PTC-100 thermo-cyclers and PCR products (10 µl) were visualized on 1.6% agarose gel and photographed using BIORAD UV-Gel documentation system using Quantity one 1-D Analysis software. The qRT-PCR analyses were performed using Applied Biosystems (AB) 7900HT Fast Real-Time PCR system 384 well format with denaturation at 95°C for 10 min, followed by 40 cycles of denaturation at 95°C for 15 s and annealing/extension at 60°C for 1 min. The amplification of an actin gene (*OsACT1*) was used as an internal control to normalize the data when real-time PCR was carried out for analyzing the expression patterns under various hormones using tissues at the same developmental stage. However, the *UBQ5* gene was used as a control when analyzing the expression profiles among different tissues since *OsACT1* gene did not show stable expression in various rice tissues as described (Jain et al., 2006).

Total RNA samples from six different rice tissues, young and old samples of roots, leaves and panicles were collected for such analyses. Young leaves and roots were collected from two week-old seedlings and young panicles were collected from booting plants. The fully erect panicles with ∼50% of opened florets were considered as mature panicles. The mature leaves and roots were collected from the same plants which were used as a source of mature panicles. On the other hand, RNA samples were also prepared from various transgenic plants for expression analysis. All the samples collected were frozen in liquid nitrogen and used immediately or stored at -80°C freezer until further use.

### Constructs and *Agrobacterium*-mediated transformation

For complementation analysis, around 4600 bp *OsCYP96B4* genomic fragment was amplified by PCR, which contains 2 Kb upstream of the start codon, 1.6 Kb coding region and 1 Kb 3′ end of genomic sequence containing the translation termination codon of *OsCYP96B4* (Figure S5). After verification by sequencing, the fragment was subcloned into pCAMBIA1300 Ti-derived binary vector (pCAMBIA; www.cambia.org.au) and was introduced into the *oscyp96b4* mutant genome by *Agrobacterium* mediated transformation. The independent transgenic plants were identified by Southern blot hybridization.

For the over-expression analysis, the maize ubiquitin promoter was employed to drive the *OsCYP96B4* expression (Figure S5). For the promoter-GFP and ectopic expression constructs, the endogenous promoter was employed as shown in Figure S5. For dsRNAi analysis, the vector pTCK303 [46] was used to prepare the construct as shown in Figure S6. *Agrobacterium-*meditated transformation protocol was modified from Hiei et al. (1994) [47]. Scutellum derived embryogenic calli from rice cv. Nipponbare was induced on callus induction medium (NB basal salts [47] with 2, 4- dichlorophenoxyacetic acid (2, 4-D) 2 mg/l) for 4 weeks at 25–28°C in dark and calli were sub-cultured in the medium with the same composition for 2 more weeks. Actively growing calluses were used as explants for transformation with *Agrobacterium tumefaciens* (*AGL1*) harboring appropriate cassettes in the binary vector pCAMBIA1300.

For the over-expression of *OsCYP96B4* in Arabidopsis, the same construct used in rice were employed (Figure S5). To generate stable transgenic lines, wild type (Col-0) Arabidopsis plants were transformed by *Agrobacterium*-mediated transformation using the floral dip method [48]. Transgenic lines were obtained on selective MS media containing hygromycin. T2 or T3 lines were used for phenotype characterization.

### Heterologous expression of *OsCYP96B4* in *S. pombe*

The *OsCYP96B4* gene was amplified by PCR using genomic DNA as template. Restriction enzyme sites *Nde*I in the 5′ and *Bam*HI in the 3′ were added in the primer sequence. The *OsCYP96B4* products were sub-cloned into *S. pombe* expression vector pREP1 (*leu* ^+^) under the *nmt1* promoter [49] and transformed into wild type *S. pombe* strain MBY192 cells. Expression was induced in minimal medium in the absence of thiamine. After 24 h of induction, the induced and un-induced cells were stained with 4′ 6,-diamidino-2-phenylindole (DAPI) and aniline blue as described by Mishra et al. (2005) [50].

### Microscopy

Leica microscope was used for the morphological observations of pollen grains and their tube germination. The investigation of cell size was carried out by measuring cell length and width under Cryo-SEM. Both GFP activity and localization of fusion protein were visualized under a confocal microscope (Zeiss, Jena, Germany).

### Lipid extraction and gas chromatography and mass spectroscopy analysis

Rice leaves were cut into small pieces (∼2 cm) and incubated in 75°C preheated IPA with 0.01% BHT and lipids were extracted following the protocol for extraction of lipids from Arabidopsis leaf tissue as specified by Kansas Lipidomics Reseach Center (KLRC), Kansas USA (http://www.k-state.edu/lipid/lipidomics/leaf-extraction.html). Dried lipid extracts were sent in dry ice to KLRC for total lipid profiling using mass spectroscopy analysis. The lipid extracts (∼3 ng) were prepared using the same protocol and were resuspended in 200 µl of hexane. The methyl estrification of fatty acids were carried out as follows. One ml of 3N methanolic HCl was added to the lipid extracts in the 30 ml glass tubes with Teflon lined caps (DAIGGER Cat. No. LC28356G) and mixed well followed by addition of 400 µl 1, 2-Dimethoxyprophane and finally 800 µl of hexane was added and mixed well. The caps were tightly sealed and the tubes were heated at 70°C for 15 min then cooled at room temperature for 15 min. The esterified fatty acids were extracted with 1 ml each of water and hexane and the upper ester layer was collected by glass pipette. This extraction procedure was repeated thrice and combined, dried under stream of N2 gas, followed by re-suspension in 100 µl hexane and finally subjected to gas chromatography analysis. Fatty acid methyl esters were analyzed by gas chromatography using a fully automated Agilent Technologies 6890N Network GC system. The chromatography utilized an Agilent 19091S-433 capillary column (30 m×250 µm×0.25 µm). Peaks were identified by comparing with fatty acid methyl ester mixer standards (Supelco, Cat. No. 18918) and, area and its percentage for each resolved peak were analyzed using Agilent Technologies GC ChemStation B, 02.01-SR2 (260) software.

## Supporting Information

Figure S1 **The effect of various hormones on the growth of both WT and** ***oscyp96b4*** **.** MS media were supplemented with different concentrations of hormones and both WT and the mutants seeds were inoculated into the MS media. Plant height was measured after 14-day growth. (A) to (E) showed the effects of ABA, IAA, KT, MeJA and SA on plant development between WT and the mutant, respectively. For the ABA treatment, very limited growth was observed at the concentration 10 µM for both WT and the mutant. For all hormone treatments, no significant difference has been observed by t-test (p>0.05) in response to different hormones between WT and the mutant.
(PPT)

Figure S2 **Genetics, revertant and complementation analyses. (A) and (B) Southern blot hybridization.** Genomic DNA samples isolated from WT (Lane 2, 4, 6, 8 and 10) and *oscyp96b4* (Lane 3, 5, 7, 9, and 11) were digested with various enzymes as shown above and were hybridized with the *GUS* probe (A) and the *OsCYP96B4* probe (B), respectively. Lane 1 and 12 in (A) and (B) are DIG-labeled DNA markers III and VII from Roche, respectively. (C) Co-segregation analysis between the mutant phenotype and *Ds* insertion using a heterozygous F2 population. (D) Sequences showing an 8-bp target duplication (red) after *Ds* insertion into the *OsCYP96B4* gene in the mutant and footprints with 1- (revertant 1), 8- (revertant 2) and 9- (revertant 3) bp of insertion (compared to WT) after *Ds* remobilization in revertants. Base substitutions in three independent revertants were shown in blue color. (E) Phenotype of WT (left), revertant 3 (middle) and revertant 1 (right). (F) Detailed investigation of florets and viable seeds in the revertant 3 by comparison with WT. (G) The copy number determination of T-DNA insertion in the complementation plants. (H) Phenotype complementation of the semi-dwarf trait in five independent transgenic plants (C1, C2, C3, C5 and C6) with single copy of T-DNA insertion (blue color) by comparing with WT (1, green color) and the mutant plants (2, red color). (I) Expression analysis of transgenic plants by quantitative real-time PCR. 1, expression level in WT plants; 2, expression level in *oscyp96b4* plants; 3, average expression level in complementation plants.
(PPT)

Figure S3 **Phenotype characterization of over-expression of rice** ***oscypb4*** **in Arabidopsis background and T-DNA insertion mutants of** ***AtCYP96A1*** **and** ***AtCYP96A10*** **.** (A) Compared with WT (left), reduced leaf size (right) was observed in transgenic Arabidopsis with over-expression of rice *OsCYP96B4*. (B) WT Arabidopsis at flowering stage (top) and transgenic flowering Arabidopsis plants over-expressing rice *OsCYP96B4* (bottom). (C) No obvious difference was observed in both T-DNA insertion mutants *atcyp96a1* and *atcyp96a10* when compared with WT. (D) Similar silique development was also observed among WT, *atcyp96a1* and *atcyp96a10*.
(PPT)

Figure S4 **Phylogenetic analysis, amino acid sequence similarity and expression profiling of tandemly duplicated CYP96 sub-family members.** (A) Phylogenetic tree constructed with P450 domain amino acid sequences from tandemly duplicated *CYP96* genes in rice, sorghum and *B. distachyon.* (B) Amino acid sequence similarity among 5 tandemly duplicated rice *CYP96B* genes. (C) Expression profiling of 5 tandemly duplicated rice *CYP96B* genes based on rice microarray analysis. The expression data are retrieved from the website: http://signal.salk.edu/cgi-bin/RiceGE?JOB=APPENDIX&QUERY=GeneAtlas.
(PPT)

Figure S5 **Constructs for promoter, ectopic/over-expression, and complementation analysis.** (A) *OsCYP96B4* promoter sGFP fusion. (B) Ectopic-expression. (C) Complementation and over-expression. (D) *OsCYP96B4* cDNA-sGFP fusion.
(PPT)

Figure S6 **Construct used for dsRNAi analyses.** The backbone vector pTCK303 is a gift from Prof. Kang Chong [46].
(PPT)

Table S1 **List of primers used for genotyping, probe synthesis, cloning and expression analysis.**
(XLS)
