# Supplementary material for: Oryza sativa Cytochrome P450 Family Member OsCYP96B4 Reduces Plant Height in a Transcript Dosage Dependent Manner
Source: PLoS One. 2011 Nov 28;6(11):e28069. doi: 10.1371/journal.pone.0028069 (PMC3225389; doi:10.1371/journal.pone.0028069)
Supplement: Figure S1 — The effect of various hormones on the growth of both WT and oscyp96b4 . MS media were supplemented with different concentrations of hormones and both WT and the mutants seeds were inoculated into the MS media. Plant height was measured after 14-day growth. (A) to (E) showed the effects of ABA, IAA, KT, MeJA and SA on plant development between WT and the mutant, respectively. For the ABA treatment, very limited growth was observed at the concentration 10 µM for both WT and the mutant. For all hormone treatments, no significant difference has been observed by t-test (p>0.05) in response to different hormones between WT and the mutant. (PPT) [file pone.0028069.s001.ppt]

## Slide 1
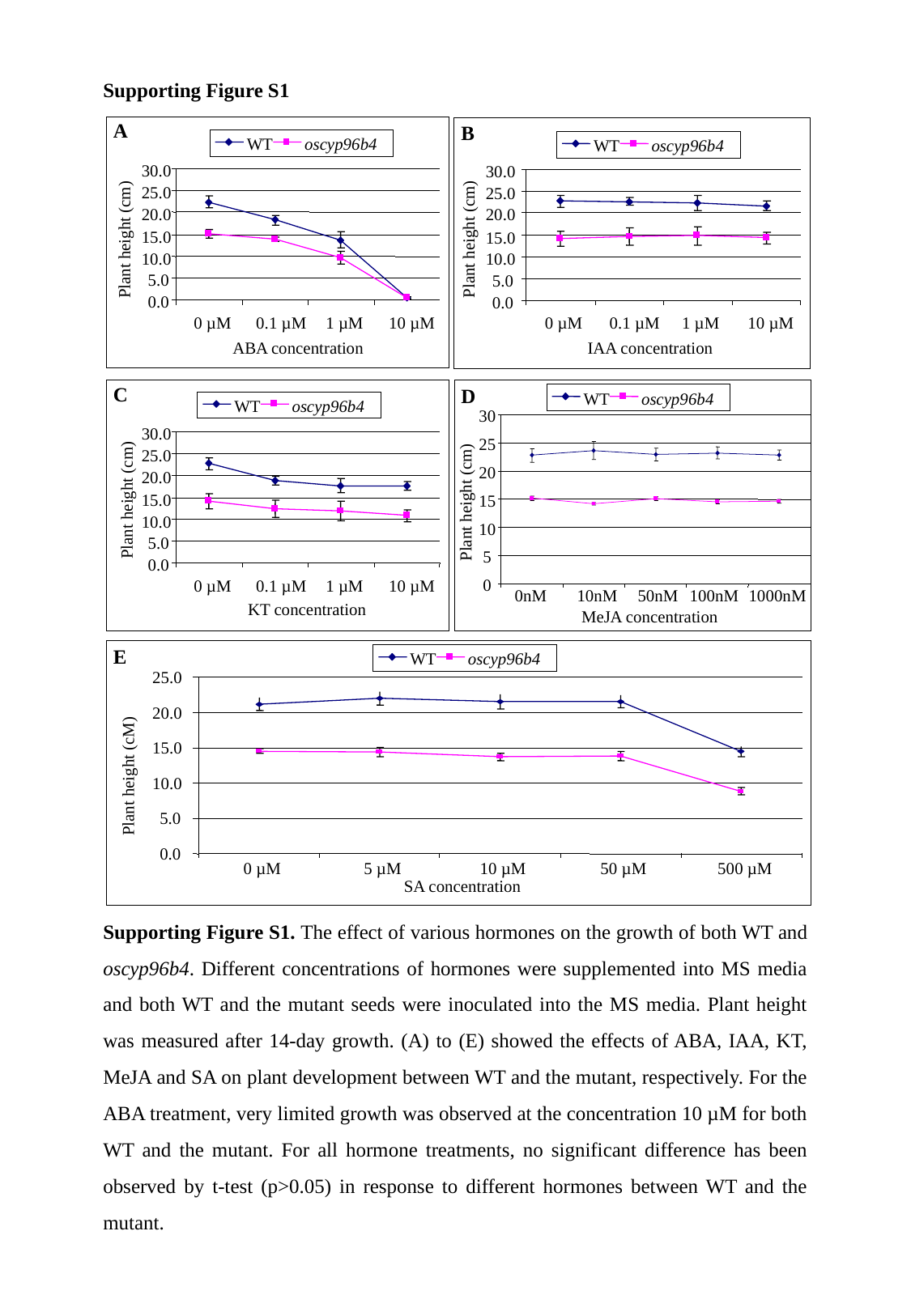

Supporting Figure S1
A
B
WT
oscyp96b4
WT
oscyp96b4
30.0
30.0
25.0
25.0
20.0
20.0
15.0
15.0
Plant height (cm)
Plant height (cm)
10.0
10.0
5.0
5.0
0.0
0.0
0 µM
0.1 µM
1 µM
10 µM
0 µM
0.1 µM
1 µM
10 µM
ABA concentration
IAA concentration
C
D
WT
oscyp96b4
WT
oscyp96b4
30
25
20
15
10
5
0
30.0
25.0
20.0
15.0
Plant height (cm)
Plant height (cm)
10.0
5.0
0.0
0 µM
0.1 µM
1 µM
10 µM
0nM
10nM
50nM
100nM
1000nM
KT concentration
MeJA concentration
E
WT
oscyp96b4
25.0
20.0
15.0
10.0
5.0
0.0
Plant height (cM)
0 µM
5 µM
10 µM
50 µM
500 µM
SA concentration
Supporting Figure S1. The effect of various hormones on the growth of both WT and oscyp96b4. Different concentrations of hormones were supplemented into MS media and both WT and the mutant seeds were inoculated into the MS media. Plant height was measured after 14-day growth. (A) to (E) showed the effects of ABA, IAA, KT, MeJA and SA on plant development between WT and the mutant, respectively. For the ABA treatment, very limited growth was observed at the concentration 10 µM for both WT and the mutant. For all hormone treatments, no significant difference has been observed by t-test (p>0.05) in response to different hormones between WT and the mutant.
