# Supplementary material for: Oryza sativa Cytochrome P450 Family Member OsCYP96B4 Reduces Plant Height in a Transcript Dosage Dependent Manner
Source: PLoS One. 2011 Nov 28;6(11):e28069. doi: 10.1371/journal.pone.0028069 (PMC3225389; doi:10.1371/journal.pone.0028069)
Supplement: Figure S2 — Genetics, revertant and complementation analyses. (A) and (B) Southern blot hybridization. Genomic DNA samples isolated from WT (Lane 2, 4, 6, 8 and 10) and oscyp96b4 (Lane 3, 5, 7, 9, and 11) were digested with various enzymes as shown above and were hybridized with the GUS probe (A) and the OsCYP96B4 probe (B), respectively. Lane 1 and 12 in (A) and (B) are DIG-labeled DNA markers III and VII from Roche, respectively. (C) Co-segregation analysis between the mutant phenotype and Ds insertion using a heterozygous F2 population. (D) Sequences showing an 8-bp target duplication (red) after Ds insertion into the OsCYP96B4 gene in the mutant and footprints with 1- (revertant 1), 8- (revertant 2) and 9- (revertant 3) bp of insertion (compared to WT) after Ds remobilization in revertants. Base substitutions in three independent revertants were shown in blue color. (E) Phenotype of WT (left), revertant 3 (middle) and revertant 1 (right). (F) Detailed investigation of florets and viable seeds in the revertant 3 by comparison with WT. (G) The copy number determination of T-DNA insertion in the complementation plants. (H) Phenotype complementation of the semi-dwarf trait in five independent transgenic plants (C1, C2, C3, C5 and C6) with single copy of T-DNA insertion (blue color) by comparing with WT (1, green color) and the mutant plants (2, red color). (I) Expression analysis of transgenic plants by quantitative real-time PCR. 1, expression level in WT plants; 2, expression level in oscyp96b4 plants; 3, average expression level in complementation plants. (PPT) [file pone.0028069.s002.ppt]

## Slide 1
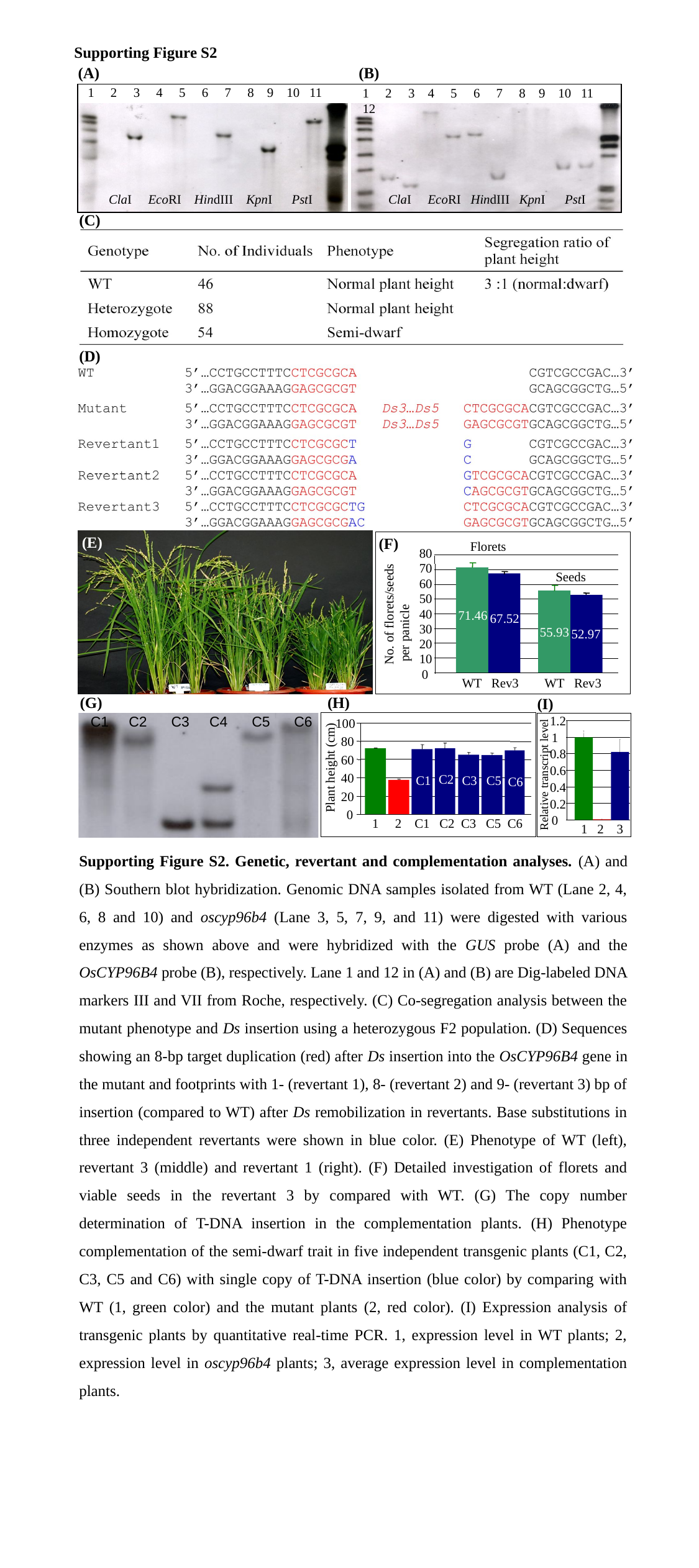

Supporting Figure S2
(A)
(B)
1 2 3 4 5 6 7 8 9 10 11 12
1 2 3 4 5 6 7 8 9 10 11 12
 ClaI EcoRI HindIII KpnI PstI
 ClaI EcoRI HindIII KpnI PstI
(C)
(D)
(E)
(F)
Florets
80
70
60
50
40
30
20
10
0
Seeds
No. of florets/seeds
 per panicle
71.46
67.52
55.93
52.97
WT Rev3 WT Rev3
(G)
(H)
(I)
 C1 C2 C3 C4 C5 C6
1.2
1
0.8
0.6
0.4
0.2
0
100
80
60
Plant height (cm)
Relative transcript level
40
C2
C1
C3
C5
C6
20
0
1 2 C1 C2 C3 C5 C6
1 2 3
Supporting Figure S2. Genetic, revertant and complementation analyses. (A) and (B) Southern blot hybridization. Genomic DNA samples isolated from WT (Lane 2, 4, 6, 8 and 10) and oscyp96b4 (Lane 3, 5, 7, 9, and 11) were digested with various enzymes as shown above and were hybridized with the GUS probe (A) and the OsCYP96B4 probe (B), respectively. Lane 1 and 12 in (A) and (B) are Dig-labeled DNA markers III and VII from Roche, respectively. (C) Co-segregation analysis between the mutant phenotype and Ds insertion using a heterozygous F2 population. (D) Sequences showing an 8-bp target duplication (red) after Ds insertion into the OsCYP96B4 gene in the mutant and footprints with 1- (revertant 1), 8- (revertant 2) and 9- (revertant 3) bp of insertion (compared to WT) after Ds remobilization in revertants. Base substitutions in three independent revertants were shown in blue color. (E) Phenotype of WT (left), revertant 3 (middle) and revertant 1 (right). (F) Detailed investigation of florets and viable seeds in the revertant 3 by compared with WT. (G) The copy number determination of T-DNA insertion in the complementation plants. (H) Phenotype complementation of the semi-dwarf trait in five independent transgenic plants (C1, C2, C3, C5 and C6) with single copy of T-DNA insertion (blue color) by comparing with WT (1, green color) and the mutant plants (2, red color). (I) Expression analysis of transgenic plants by quantitative real-time PCR. 1, expression level in WT plants; 2, expression level in oscyp96b4 plants; 3, average expression level in complementation plants.
