# Supplementary material for: Oryza sativa Cytochrome P450 Family Member OsCYP96B4 Reduces Plant Height in a Transcript Dosage Dependent Manner
Source: PLoS One. 2011 Nov 28;6(11):e28069. doi: 10.1371/journal.pone.0028069 (PMC3225389; doi:10.1371/journal.pone.0028069)
Supplement: Figure S3 — Phenotype characterization of over-expression of rice oscypb4 in Arabidopsis background and T-DNA insertion mutants of AtCYP96A1 and AtCYP96A10 . (A) Compared with WT (left), reduced leaf size (right) was observed in transgenic Arabidopsis with over-expression of rice OsCYP96B4. (B) WT Arabidopsis at flowering stage (top) and transgenic flowering Arabidopsis plants over-expressing rice OsCYP96B4 (bottom). (C) No obvious difference was observed in both T-DNA insertion mutants atcyp96a1 and atcyp96a10 when compared with WT. (D) Similar silique development was also observed among WT, atcyp96a1 and atcyp96a10. (PPT) [file pone.0028069.s003.ppt]

## Slide 1
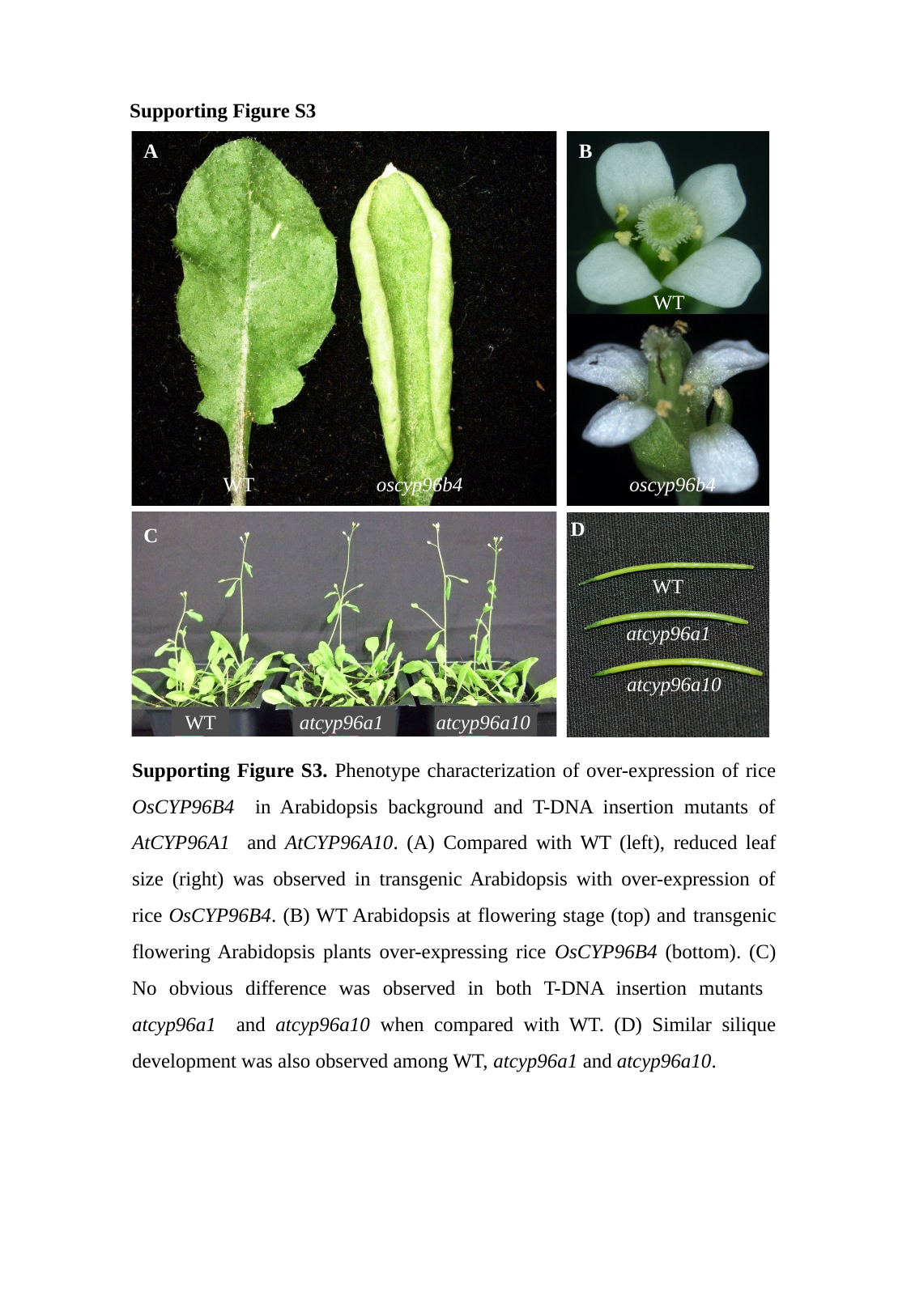

Supporting Figure S3
A
B
WT
WT
oscyp96b4
oscyp96b4
D
C
WT
atcyp96a1
atcyp96a10
WT
atcyp96a1
atcyp96a10
Supporting Figure S3. Phenotype characterization of over-expression of rice OsCYP96B4 in Arabidopsis background and T-DNA insertion mutants of AtCYP96A1 and AtCYP96A10. (A) Compared with WT (left), reduced leaf size (right) was observed in transgenic Arabidopsis with over-expression of rice OsCYP96B4. (B) WT Arabidopsis at flowering stage (top) and transgenic flowering Arabidopsis plants over-expressing rice OsCYP96B4 (bottom). (C) No obvious difference was observed in both T-DNA insertion mutants atcyp96a1 and atcyp96a10 when compared with WT. (D) Similar silique development was also observed among WT, atcyp96a1 and atcyp96a10.
