# Supplementary material for: Oryza sativa Cytochrome P450 Family Member OsCYP96B4 Reduces Plant Height in a Transcript Dosage Dependent Manner
Source: PLoS One. 2011 Nov 28;6(11):e28069. doi: 10.1371/journal.pone.0028069 (PMC3225389; doi:10.1371/journal.pone.0028069)
Supplement: Figure S4 — Phylogenetic analysis, amino acid sequence similarity and expression profiling of tandemly duplicated CYP96 sub-family members. (A) Phylogenetic tree constructed with P450 domain amino acid sequences from tandemly duplicated CYP96 genes in rice, sorghum and B. distachyon. (B) Amino acid sequence similarity among 5 tandemly duplicated rice CYP96B genes. (C) Expression profiling of 5 tandemly duplicated rice CYP96B genes based on rice microarray analysis. The expression data are retrieved from the website: http://signal.salk.edu/cgi-bin/RiceGE?JOB=APPENDIX&QUERY=GeneAtlas. (PPT) [file pone.0028069.s004.ppt]

## Slide 1
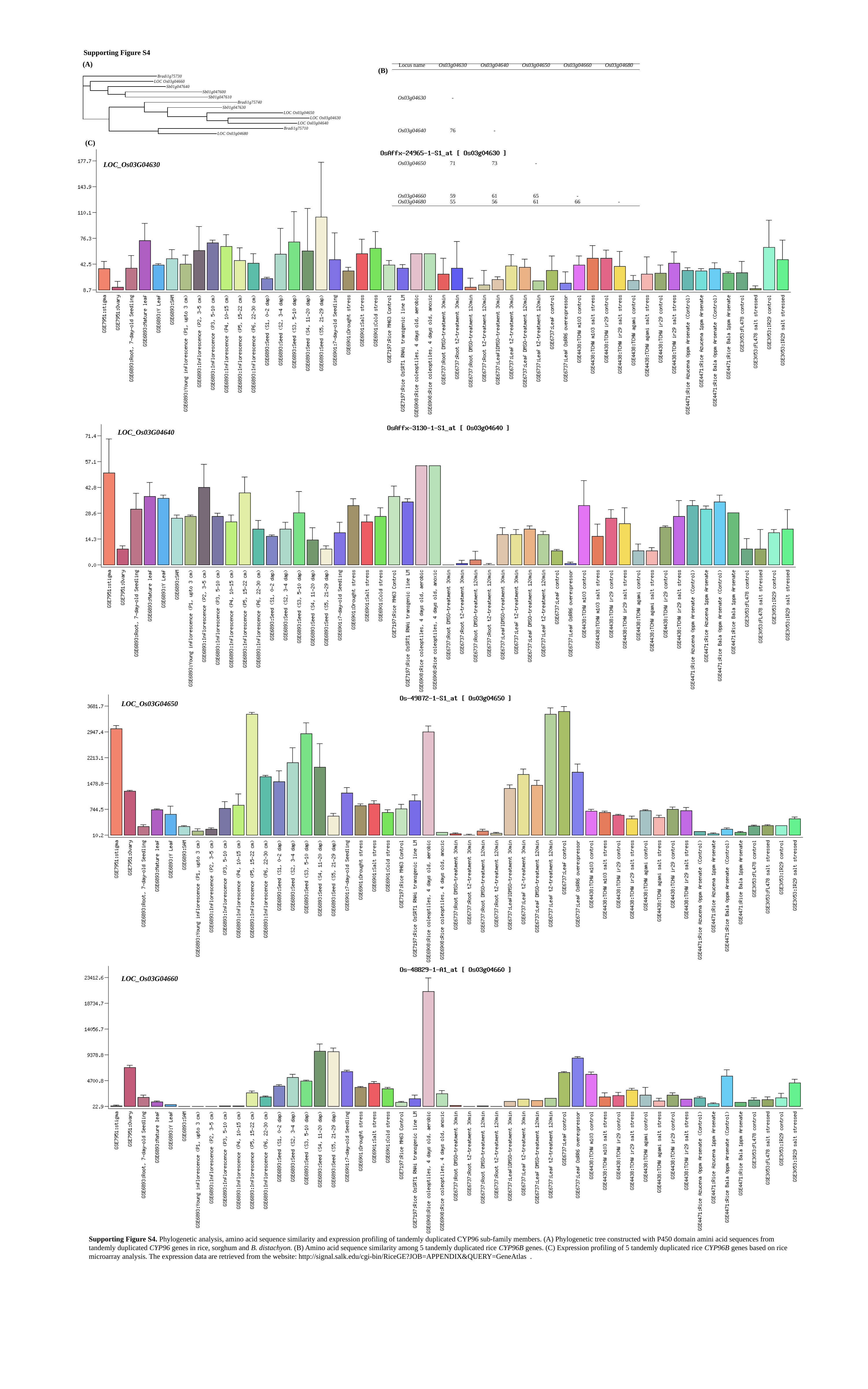

Supporting Figure S4
(A)
(B)
| Locus name | Os03g04630 | Os03g04640 | Os03g04650 | Os03g04660 | Os03g04680 |
| --- | --- | --- | --- | --- | --- |
| Os03g04630 | - | | | | |
| Os03g04640 | 76 | - | | | |
| Os03g04650 | 71 | 73 | - | | |
| Os03g04660 | 59 | 61 | 65 | - | |
| Os03g04680 | 55 | 56 | 61 | 66 | - |
(C)
LOC_Os03G04630
LOC_Os03G04640
LOC_Os03G04650
LOC_Os03G04660
Supporting Figure S4. Phylogenetic analysis, amino acid sequence similarity and expression profiling of tandemly duplicated CYP96 sub-family members. (A) Phylogenetic tree constructed with P450 domain amini acid sequences from tandemly duplicated CYP96 genes in rice, sorghum and B. distachyon. (B) Amino acid sequence similarity among 5 tandemly duplicated rice CYP96B genes. (C) Expression profiling of 5 tandemly duplicated rice CYP96B genes based on rice microarray analysis. The expression data are retrieved from the website: http://signal.salk.edu/cgi-bin/RiceGE?JOB=APPENDIX&QUERY=GeneAtlas .
