# Supplementary material for: Oryza sativa Cytochrome P450 Family Member OsCYP96B4 Reduces Plant Height in a Transcript Dosage Dependent Manner
Source: PLoS One. 2011 Nov 28;6(11):e28069. doi: 10.1371/journal.pone.0028069 (PMC3225389; doi:10.1371/journal.pone.0028069)
Supplement: Figure S5 — Constructs for promoter, ectopic/over-expression, and complementation analysis. (A) OsCYP96B4 promoter sGFP fusion. (B) Ectopic-expression. (C) Complementation and over-expression. (D) OsCYP96B4 cDNA-sGFP fusion. (PPT) [file pone.0028069.s005.ppt]

## Slide 1
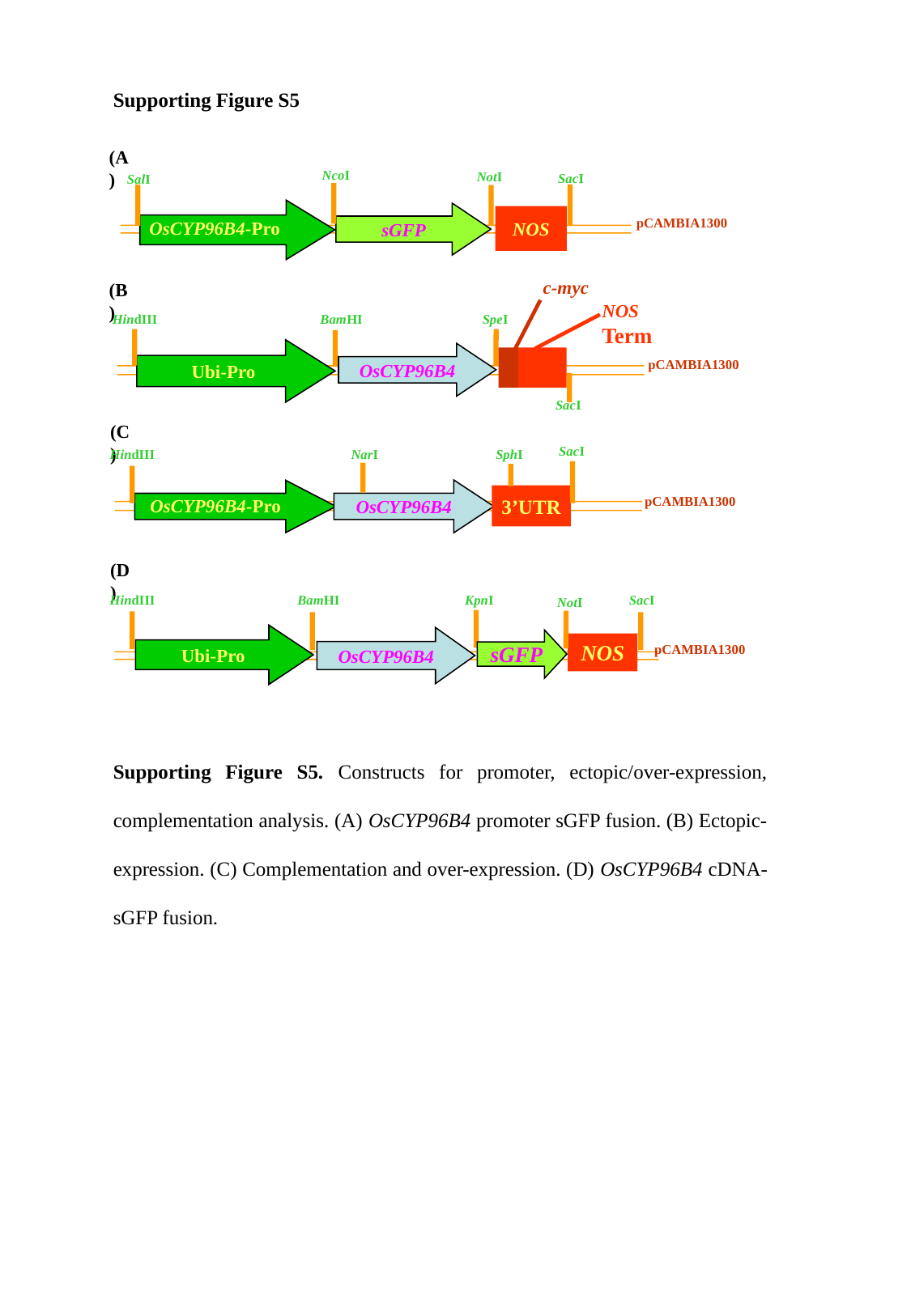

Supporting Figure S5
(A)
NcoI
NotI
SacI
SalI
sGFP
NOS
pCAMBIA1300
OsCYP96B4-Pro
c-myc
NOS Term
HindIII
BamHI
SpeI
Ubi-Pro
OsCYP96B4
pCAMBIA1300
SacI
(B)
(C)
SacI
NarI
HindIII
SphI
OsCYP96B4
3’UTR
pCAMBIA1300
OsCYP96B4-Pro
(D)
SacI
HindIII
BamHI
KpnI
NotI
Ubi-Pro
OsCYP96B4
sGFP
NOS
pCAMBIA1300
Supporting Figure S5. Constructs for promoter, ectopic/over-expression, complementation analysis. (A) OsCYP96B4 promoter sGFP fusion. (B) Ectopic-expression. (C) Complementation and over-expression. (D) OsCYP96B4 cDNA-sGFP fusion.
