# Supplementary material for: Oryza sativa Cytochrome P450 Family Member OsCYP96B4 Reduces Plant Height in a Transcript Dosage Dependent Manner
Source: PLoS One. 2011 Nov 28;6(11):e28069. doi: 10.1371/journal.pone.0028069 (PMC3225389; doi:10.1371/journal.pone.0028069)
Supplement: Figure S6 — Construct used for dsRNAi analyses. The backbone vector pTCK303 is a gift from Prof. Kang Chong [46]. (PPT) [file pone.0028069.s006.ppt]

## Slide 1
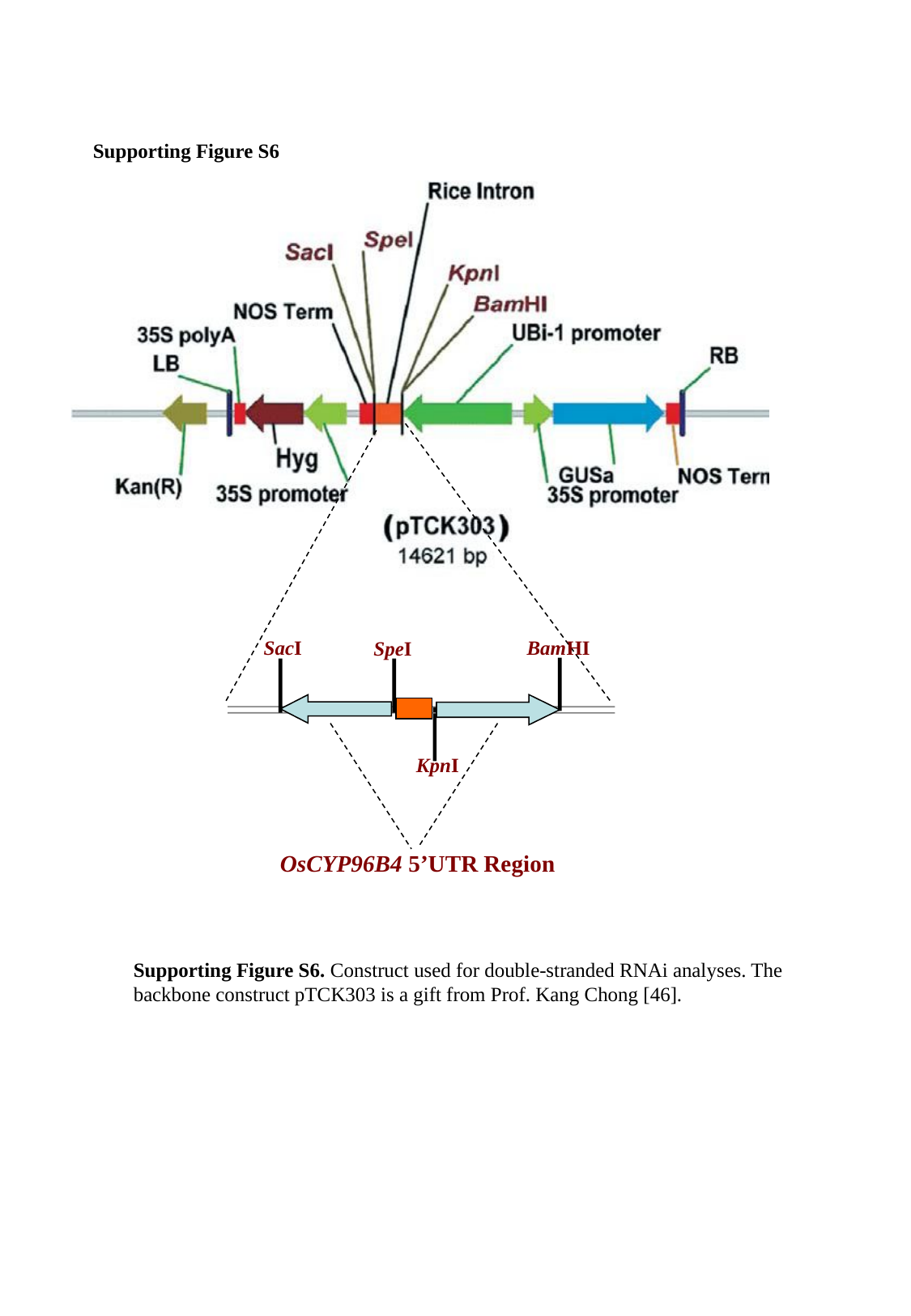

SacI
BamHI
SpeI
KpnI
OsCYP96B4 5’UTR Region
Supporting Figure S6
Supporting Figure S6. Construct used for double-stranded RNAi analyses. The backbone construct pTCK303 is a gift from Prof. Kang Chong [46].
